# Baixo peso ao nascer, prematuridade e restrição de crescimento intra-uterino: resultados dos dados de base da primeira coorte de nascimentos indígenas no Brasil (coorte de nascimentos Guarani)

**DOI:** 10.1186/s12884-020-03396-8

**Published:** 2020-12-02

**Authors:** Carla Tatiana Garcia Barreto, Felipe Guimarães Tavares, Mariza Theme-Filha, Yasmin Nascimento Farias, Lídia de Nazaré Pantoja, Andrey Moreira Cardoso

**Affiliations:** 1 Universidade do Estado do Rio de Janeiro (UERJ), Av. Marechal Rondon, 381. São Francisco Xavier, Rio de Janeiro, RJ CEP: 20950-000 Brasil; 2 Escola Nacional de Saúde Pública, Fundação Oswaldo Cruz, Rio de Janeiro, RJ Brasil; 3 Escola de Enfermagem Aurora de Afonso Costa. Faculdade de Enfermagem, Universidade Federal Fluminense, Niterói, RJ Brasil

**Keywords:** População indigena, C saúde das hildren, Baixo peso de nascimento, Nascimento prematuro, Restrição de crescimento intrauterino, Prevalência

## Abstract

**fundo:**

O baixo peso ao nascer (BPN) continua sendo um importante problema de saúde global, associado a uma série de resultados adversos de saúde ao longo da vida. As evidências sugerem que o BPN é um determinante relevante de morbidade e mortalidade em grupos indígenas, que geralmente têm acesso limitado às políticas públicas de saúde e nutrição. O conhecimento da prevalência de BPN e de suas causas subjacentes pode contribuir com etapas essenciais para a prevenção de seus efeitos sobre a saúde. O estudo teve como objetivo estimar as prevalências de BPN, prematuridade e restrição de crescimento intra-uterino (RCIU) e investigar seus determinantes na primeira coorte de nascimentos indígenas no Brasil.

**Métodos:**

Este estudo transversal utilizou dados de linha de base coletados da primeira coorte de nascimentos indígenas no Brasil, a Coorte de Nascimentos Guarani. O Brasil é um dos países com maior diversidade étnica do mundo, com 305 povos indígenas e 274 línguas nativas. Os Guarani são uma das cinco maiores etnias, com aldeias localizadas principalmente na região sul. Todos os nascimentos únicos de 1º de junho de 2014 a 31 de maio de 2016 foram selecionados em 63 aldeias indígenas Guarani nas regiões Sul e Sudeste. Foi realizada regressão logística múltipla hierárquica.

**Resultados:**

As taxas de prevalência de BPN, prematuridade e RCIU foram 15,5, 15,6 e 5,7%, respectivamente. As chances de BPN foram menores em recém-nascidos de mães que vivem em casas de tijolo e argamassa (OR: 0,25; IC 95%: 0,07-0,84) e foram maiores em filhos de mães ≤20 anos de idade (OR: 2,4; IC 95%: 1,29-4,44) e com anemia crônica antes da gravidez (OR: 6,41; IC 95%: 1,70-24,16). A prematuridade foi estatisticamente associada ao tipo de fonte de energia para cozinhar (fogão a lenha - OR: 3,87; IC 95%: 1,71–8,78 e fogueiras - OR: 2,57; IC 95%: 1,31–5,01). RCIU foi associado à primiparidade (OR: 4,66; IC 95%: 1,68–12,95) e anemia materna crônica antes da gravidez (OR: 7,21; IC 95%: 1,29–40,38).

**Conclusões:**

Idade materna, estado nutricional e paridade, condições de moradia e exposição à poluição interna foram associados com resultados perinatais na população indígena Guarani. Esses resultados indicam a necessidade de investir no acesso e melhoria da assistência pré-natal; também no fortalecimento do Subsistema de Saúde Indígena, e em ações intersetoriais para o desenvolvimento de políticas habitacionais e de saneamento e melhorias ambientais ajustadas às necessidades e conhecimentos dos povos indígenas.

**Informação suplementar:**

**Informações suplementares** acompanham este documento em 10.1186/s12884-020-03396-8 .

## fundo

O baixo peso ao nascer (BPN) continua sendo um problema de saúde global altamente relevante, associado a uma série de resultados adversos de saúde ao longo da vida [1]. O peso ao nascer pode ser determinado tanto pelo crescimento fetal quanto pela duração da gravidez [2]. Crianças com BPN apresentam risco aumentado de infecções [3, 4], atrasos no crescimento e no desenvolvimento físico e cognitivo [5] e mortalidade [6]. As repercussões do BPN duram até a idade adulta, conferindo maior risco de doenças crônicas não transmissíveis e mortalidade por todas as causas e cardiovascular [7] Além disso, estudos relatam efeitos socioeconômicos diretos, evidenciados pela associação com menores níveis de escolaridade, menores taxas de emprego e maior dependência de benefícios sociais [8].

A prevalência global estimada de BPN ultrapassa 15%, correspondendo a cerca de 20 milhões de nascimentos. Embora essa taxa seja alta, parece ter sido subestimada, uma vez que mais de 95% dos casos de BPN ocorrem em países em desenvolvimento e nas populações mais vulneráveis, onde há uma proporção maior de partos em casa e dados menos confiáveis ​​registrados sobre o peso ao nascer [1] . A identificação de populações com risco aumentado de BPN e que enfrentam barreiras para acessar políticas de saúde e nutrição são, portanto, uma prioridade de saúde global [1]. Além disso, identificar a contribuição da prematuridade e da restrição de crescimento intrauterino (RCIU) para o BPN e suas causas subjacentes pode fornecer etapas essenciais para a prevenção dessa condição e de seus efeitos sobre a saúde.

Os povos indígenas são considerados populações marginalizadas e em grande parte experimentam baixos padrões de saúde [9–11]. Anderson et al. [9] compararam os indicadores socioeconômicos e de saúde de 28 povos indígenas em 23 países com os respectivos indicadores de populações de referência não indígenas e mostraram uma desvantagem sistemática para os povos indígenas, incluindo a prevalência de BPN. Apesar da vasta literatura internacional sobre BPN, são poucos os estudos relacionados aos povos indígenas, principalmente na América Latina como um todo e no Brasil [12], país com uma das populações indígenas mais diversificadas socialmente do mundo [13].

Uma revisão sistemática recente [12] constatou que os fatores de risco para BPN em povos indígenas são semelhantes aos identificados em populações não indígenas com baixo nível socioeconômico. Estas incluíram causas obstétricas como primiparidade [14–16] e história de prematuridade [15, 17–19], natimorto [16, 18] ou aborto [16], hipertensão induzida pela gravidez [15, 17, 19] e infecção urinária durante a gravidez [15, 17, 19]. Os autores também encontraram fatores de risco relacionados ao estado nutricional materno, incluindo baixo ganho de peso gestacional [17], desnutrição pré-gestacional [18] e anemia [15], bem como acesso limitado aos serviços de saúde [20, 21]. Múltiplos determinantes de BPN, conforme descritos na literatura, coexistem entre os povos indígenas no Brasil, além de uma alta carga de resultados adversos à saúde geralmente associados ao BPN, como hospitalização infantil e morte por infecção respiratória aguda, diarreia e desnutrição [22–28]. Essas evidências sugerem que o BPN pode ser um determinante relevante da morbimortalidade infantil em grupos indígenas no Brasil.

De acordo com o censo nacional mais recente, realizado em 2010, havia 896,9 mil indígenas no Brasil, correspondendo a 0,4% da população nacional [13]. Destes, aproximadamente 85.000 são Guarani, ou seja, 9,5% da população indígena. Os Guarani são divididos em três subgrupos étnicos baseados em especificidades religiosas, linguísticas e culturais - os Kaiowa, os Nhandéva e os Mbya. Os Mbya têm a menor população, cerca de 25.000 pessoas, um terço (8.000) das quais vive na costa sul [13, 29]. A faixa litorânea faz parte do território tradicional da população Mbya e embora nossos estudos tenham sido realizados com todos os Guarani que vivem nesta localidade, a grande maioria deles são Mbya.

Nos últimos anos, estudos epidemiológicos sobre a saúde de crianças Guarani nesta área relataram altas taxas e proporções de hospitalização [30] e morte [31] por infecções agudas do trato respiratório inferior em crianças menores de cinco anos, especialmente em bebês. As taxas têm superado as estimativas correspondentes entre outras etnias indígenas e não indígenas. O BPN foi um fator de risco independente para hospitalização de crianças Guarani por infecções agudas do trato respiratório inferior em um estudo de caso-controle prospectivo de base populacional [23] Além disso, resultados da Primeira Pesquisa Nacional de Saúde e Nutrição Indígena do Brasil relataram maior prevalência de pneumonia em crianças da região sul, que foi independentemente associada ao BPN [22]. Com o objetivo de gerar evidências científicas robustas sobre os determinantes da saúde infantil Guarani, visando reduzir a morbimortalidade por causas evitáveis, e com foco nas infecções respiratórias agudas, realizamos recentemente o primeiro estudo de uma coorte de nascimentos indígenas no Brasil - o Coorte de Nascimentos Guarani.

O objetivo deste estudo foi estimar as taxas de prevalência de BPN, prematuridade e RCIU, investigar seus determinantes e caracterizar a contribuição relativa da prematuridade e RCIU para o BPN nos dados basais da Coorte de Nascimentos Guarani.

## Métodos

Este estudo transversal utilizou dados de linha de base coletados da Coorte de Nascimentos Guarani; os participantes foram recrutados de 1º de junho de 2014 a 31 de maio de 2016.

Desde 1999, o Brasil implantou o Subsistema de Saúde Indígena (SASI-SUS) como parte do Sistema Único de Saúde (SUS). O SASI-SUS está organizado em 34 Distritos Sanitários Sanitários Indígenas, cada um deles com Equipes Multidisciplinares de Saúde Indígena (EMSI). Essas equipes são responsáveis ​​pela atenção básica às aldeias, coordenando o atendimento e vinculando o Subsistema aos demais níveis de complexidade da assistência do SUS, garantindo a integralidade da saúde indígena. Em relação à gravidez e ao parto, os enfermeiros e médicos da atenção básica são responsáveis ​​pelo pré-natal nas aldeias, com base em protocolos do Ministério da Saúde e as gestantes de alto risco são encaminhadas para especialistas em obstetrícia do SUS nos municípios próximos.

O estudo da Coorte de Nascimentos Guarani foi implementado em colaboração com os dois Distritos Sanitários Indígenas existentes no Sul e Sudeste do Brasil, a fim de investigar os determinantes da saúde infantil nos Guarani [32] A população investigada vive em vilas e acampamentos, principalmente na faixa litorânea da região sul, cercada pelas maiores cidades do Brasil, expondo-a a condições de vida desfavoráveis, como territórios pequenos, degradados, poluídos e populosos. A maioria das aldeias Guarani possui algum grau de acesso ao sistema municipal de saúde das cidades circunvizinhas, e o SASI-SUS oferece apoio quando a população utiliza serviços externos, por exemplo, transporte ou alimentação. A maioria das aldeias possui parteiras indígenas que realizam partos domiciliares, o que representa 28,5% dos partos na coorte Guarani.

**Fig. 1:**
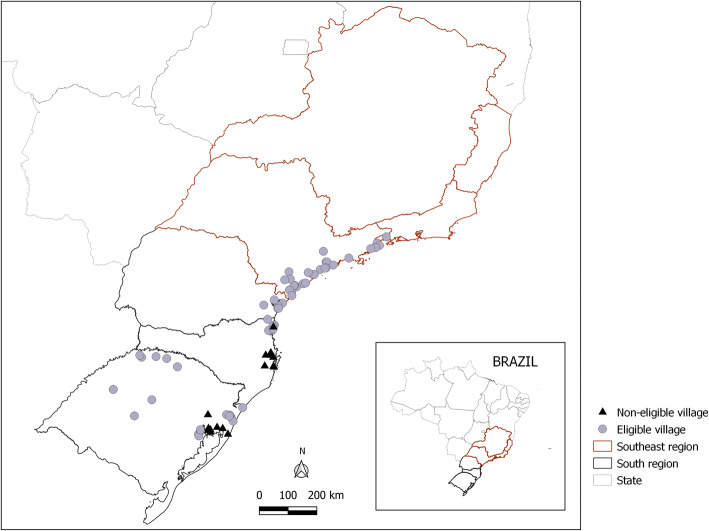
Geographic Location of Villages and Indigenous Lands in the South and Southeast of Brazil

Participants in the study were recruited from 63 villages (75.9%) of the 83 existing Guarani villages on the coastline extending from the state of Rio de Janeiro to the state of Santa Catarina and the entire state of Rio Grande do Sul (Fig. 1). Eligible villages for the birth cohort were defined as those with a structure that would allow us to implement a surveillance system for the study itself, aimed at providing weekly home follow-up of the children recruited during their first year of life. This would enable us to capture several health outcomes of interest, including vital events, incident episodes of acute short duration diseases, like acute respiratory infections and diarrhea, and the perinatal outcomes specifically analyzed in this study – LBW, prematurity and IUGR. The study aimed to include all liveborn singleton children of Guarani mothers in the eligible villages during the Guarani Cohort recruitment period. The mother was defined as Guarani if she lived in an eligible village and had reported her own indigenous ethnicity at the time of the recruitment interview.

### Operationalization of the study

The data for analysis were gathered from the perinatal questionnaire of the Guarani Birth Cohort, which was applied to the mother during postpartum (ideally within 15 days of giving birth) by previously-trained nurses from the Multidisciplinary Indigenous Health Team (EMSI).

The perinatal questionnaire was designed by the research team and was based on questionnaires from the National Census, the First National Survey of Indigenous People’s Health and Nutrition in Brazil, a population-based case-control study in the Guarani population, and other Brazilian reference studies on infant-maternal health, such as the four Pelotas Birth Cohorts (1982, 1993, 2004 and 2015) and the Birth in Brazil Study. We also added questions based on the forms routinely used in health services for prenatal care, the hospital discharge summary after birth, the health card of the child, and the birth certificate. If necessary, items were adapted to the local context, for example adding questions to measure specific variables related to the outcomes of interest in the cohort as a whole, such as household characteristics, sanitation, type and location of cooking and heating fire and additional sources of income.

O questionário (Arquivo suplementar 1) era composto por 102 questões e algumas subquestões dependentes, que foram organizadas em blocos a serem preenchidos durante a entrevista com a mãe (33 itens). Algumas informações foram extraídas de registros secundários (69 itens), para evitar entrevistas desnecessariamente longas para o participante. O tempo total para preenchimento do questionário foi de cerca de uma hora, incluindo uma entrevista de 30 minutos.

As respostas às perguntas da entrevista foram coletadas por meio de entrevista face a face com as mães ou, alternativamente, com outro parente da criança. O questionário foi elaborado em formato digital, operado em um PDA (Personal Digital Assistant) de mão, com posterior transmissão online dos dados aos coordenadores do estudo. Diversas estratégias de controle de qualidade foram adotadas para garantir o melhor preenchimento dos questionários; no entanto, alguns registros estavam incompletos devido à falta de informações nas fontes secundárias disponíveis ou porque os entrevistadores não receberam uma resposta do entrevistado. Para minimizar a perda de dados e garantir a qualidade da informação, visitas periódicas aos postos de saúde da aldeia foram agendadas pelos coordenadores do estudo para extrair dados secundários registrados nos prontuários das mães e bebês, cartões de pré-natal,

### Resultados do estudo

Três desfechos-alvo foram considerados: (1) baixo peso ao nascer - BPN; (2) prematuridade; e (3) restrição de crescimento intrauterino - RCIU. Os dados usados ​​para estimar os três desfechos foram extraídos pelos entrevistadores das seguintes fontes secundárias: certidão de nascimento, livreto de saúde da criança e prontuário hospitalar ou ambulatorial.

BPN foi definido como peso ao nascer <2.500 g. A idade gestacional ao nascimento foi calculada pelo seguinte algoritmo: estimativa diretamente a partir de laudo de ultrassom emitido em qualquer momento da gestação; na ausência de tal laudo, utilizar o registro da idade gestacional no prontuário da mãe ou no cartão de pré-natal, com base na ultrassonografia informada pelo médico ou enfermeiro, ou, alternativamente, com base na data da última menstruação (DLM) registrada no prontuário materno ou cartão de pré-natal [33]. A idade gestacional ao nascimento foi categorizada em prematura ou a termo. A prematuridade foi definida como a idade gestacional ao nascimento <37 semanas.

Calculamos o IUGR usando o programa INTE RGROWTH-21st. RCIU foi definido como escore Z de peso ao nascer para idade gestacional ≤ - 2 desvios-padrão (DP), de acordo com o Consórcio Internacional de Crescimento Fetal e Recém-nascido para o Século XXI (INTE RGROWTH-21st) [34].

### Variáveis ​​de exposição

#### Socio-econômico

“Renda regular domiciliar per capita no último mês” foi calculada como a soma dos salários do trabalho formal, transferências condicionais de dinheiro (*Bolsa Família*), benefícios de aposentadoria e pensões de todos os residentes com 10 anos ou mais de idade no mês anterior, divididos pelo número de residentes no domicílio. Esta variável foi categorizada em níveis de pobreza definidos pelo Banco Mundial com base na renda per capita diária em dólares: acima da linha de pobreza ≥ US $ 5,50/dia; pobreza, <US $ 5,50/dia a US $ 1,90/dia; e pobreza extrema ≤ US $ 1,90/dia [35].

#### Características domésticas

“Habitação de tijolo e argamassa” foi definida pela combinação de materiais de construção usados ​​para o piso, paredes e telhado, como “sim” (paredes de tijolos, ladrilhos ou piso de cimento e zinco, amianto ou telhas de cerâmica) ou “ não ”(toras, madeira, palha, barro e outros); e “uso de cobertura adicional nas paredes ou teto”, categorizada em “não” ou “sim” (uso de lonas, lonas plásticas, cobertores ou tecido para aumentar a durabilidade da casa e proteger os moradores das intempéries).

#### Variáveis ​​reprodutivas, acesso a cuidados pré-natais e morbidade materna

A adequação da assistência pré-natal foi avaliada por dois indicadores, conforme proposto por Domingues et al. [36] O indicador (1) foi o acesso ao pré-natal adequado, quando a consulta de pré-natal ocorreu antes do quarto mês de gestação (16 semanas) e o número de consultas correspondeu à recomendação para a idade gestacional no final da gravidez. O indicador (2) foi o acesso ao pré-natal quando as condições especificadas no indicador 1 foram atendidas e os exames laboratoriais de rotina foram realizados pelo menos uma vez durante a gravidez (tipo sanguíneo, hemoglobina/hematócrito, glicemia, VDRL, teste de HIV e simples teste de urina ou cultura de urina) e vacinação antitetânica materna, de acordo com o esquema recomendado. Uma vez que a entrevista perinatal foi idealmente realizada dentro de 15 dias após o parto e a consulta de acompanhamento pós-parto pode ser agendada até 42 dias após o parto, a consulta pós-parto não foi considerada na definição de cuidado pré-natal adequado [36]. O indicador 2 é, portanto, um teste de adequação mais rigoroso do que o indicador 1.

Hipertensão pré-gestacional e hipertensão gestacional foram agregadas na variável “hipertensão materna”. Os dados sobre “hipertensão materna”, “infecção urinária na gestação” e “anemia materna crônica” antes da gestação foram obtidos nos cartões de pré-natal ou prontuários das pacientes.

O estado nutricional pré-gestacional foi classificado de acordo com o IMC (peso/altura ^2^) com base no peso apenas antes da 13ª semana de gestação. O IMC pré-gestacional foi classificado como baixo peso (<18,5 kg/m ^2^), peso normal (18,5–24,9 kg/m ^2^), sobrepeso (25,0–29,9 kg/m ^2^) e obesidade (≥ 30 kg/m ^2^). Para fins de análise, o sobrepeso e a obesidade foram agrupados em uma categoria, excesso de peso (IMC ≥ 25,0 kg/m ^2^).

O ganho de peso gestacional foi calculado com base nas recomendações do Institute of Medicine de acordo com o IMC pré-gestacional: baixo peso - um ganho de 12,5–18 kg; peso normal - ganho de 11,5–16 kg; sobrepeso - um ganho de 7-11,5 kg; e obeso - um ganho de 5-9 kg. Dividimos o ganho de peso gestacional em três categorias: baixo, se o ganho de peso estava abaixo do recomendado; adequado se estivesse dentro da recomendação; e excessivo, se estiver acima da recomendação. O ganho de peso gestacional total foi ajustado para a duração da gestação no momento em que o peso final foi coletado [37].

#### Hábitos maternos durante a gravidez

“Fumar durante a gravidez” foi definido como fumar pelo menos um cigarro feito em fábrica por dia durante a gravidez, classificado como sim ou não. “Consumo de álcool na gestação” foi definido como consumo de álcool por pelo menos um trimestre, independente do período da gestação, número de doses ou tipo de bebida, categorizado em sim ou não.

Variáveis ​​adicionais sobre características do domicílio, características maternas, variáveis ​​reprodutivas e tipo de parto foram consideradas autoexplicativas e são apresentadas apenas nas tabelas. Apenas os partos hospitalares foram considerados no cálculo da taxa de cesárea.

### Análise de dados

Como primeira etapa da análise, foram excluídos os valores extremos das variáveis ​​que compunham os desfechos de interesse considerados implausíveis. Esse foi o caso para idade gestacional <20 semanas e> 44 semanas e escore Z de peso ao nascer para idade gestacional <- 4 DP ou> 4 DP, ambos usados ​​para estimativas de RCIU.

Uma análise descritiva comparativa foi feita entre nascimentos recrutados e não recrutados de acordo com algumas variáveis, incluindo taxas dos resultados alvo do estudo, seguida por uma análise descritiva das taxas de nascimentos recrutados e as taxas de prevalência para os resultados BPN, prematuridade e RCIU de acordo com as categorias de variáveis ​​independentes.

**Figura 2:**
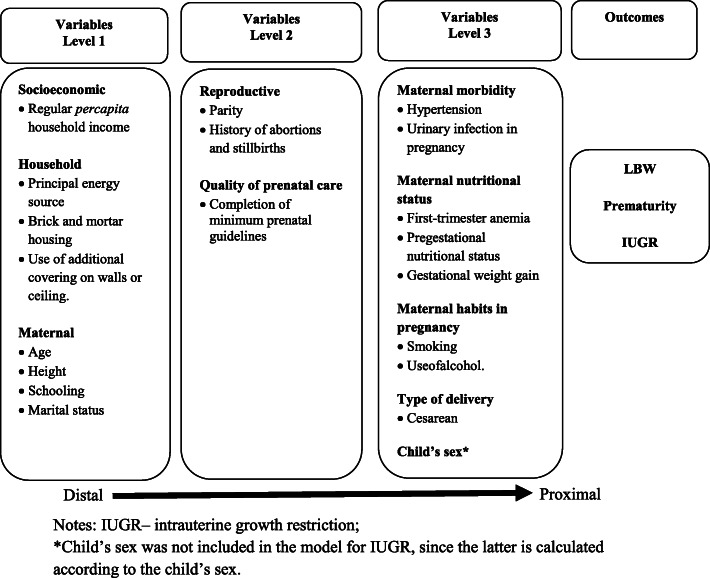
Modelo teórico hierárquico de determinação do BPN, prematuridade e restrição de crescimento intrauterino na população Guarani no Sul e Sudeste do Brasil

Foram analisadas as associações brutas entre as variáveis ​​independentes e os desfechos, estimando-se os odds ratios (OR) brutos com respectivos intervalos de confiança de 95% (IC95%) por meio de regressão logística. Para a análise multivariada, optou-se pela regressão logística hierárquica utilizando um modelo teórico de determinação do desfecho baseado no modelo originalmente proposto por Victora et al. [38] e adaptado às características da população em estudo (fig. 2).

Análises separadas foram realizadas para cada um dos três resultados-alvo do estudo. A ordem de entrada dos blocos de variáveis ​​no modelo de regressão multivariada foi definida pelos níveis hierárquicos do modelo teórico, do nível mais distal (nível 1) ao mais proximal (nível 3). O nível 1 foi composto por variáveis ​​socioeconômicas, domiciliares e características maternas. O nível 2 consistiu nas variáveis ​​reprodutivas e qualidade da assistência pré-natal e o nível 3 consistiu na morbidade e nutrição materna, hábitos maternos durante a gravidez, tipo de parto e sexo da criança.

As variáveis ​​do nível 1 que atingiram significância *p*  <0,20 na regressão logística simples foram incluídas conjuntamente no modelo de regressão multivariada pertencente a este nível, adotando-se o procedimento backward para exclusão stepwise das variáveis ​​de menor significância estatística, até o modelo final em este nível incluiu apenas variáveis ​​com significância de *p*  <0,05.

Para cada variável de nível 2, estimamos o OR ajustado pelas variáveis ​​significativas mantidas no nível 1. As variáveis ​​do nível 2 que alcançaram significância em *p*  <0,20 foram incluídas conjuntamente no modelo de regressão múltipla para este nível, juntamente com as variáveis mantidas em o modelo final do nível anterior (nível 1). O procedimento backward foi então utilizado com exclusão stepwise das variáveis ​​de menor significância estatística até que o modelo final neste nível apenas mantivesse as variáveis ​​com significância em *p*  <0,05, ajustadas pelas variáveis ​​mantidas no nível 1 e ajustadas mutuamente pelas variáveis ​​mantidas no nível 2.

Para o nível 3, os procedimentos analíticos foram realizados conforme descrito para o nível anterior até que o modelo final de determinação para cada um dos resultados alvo fosse alcançado.

O sexo da criança não foi utilizado como variável no modelo para RCIU, uma vez que esse indicador é calculado separadamente para meninos e meninas.

O programa R versão 3.4.2 foi usado para as análises [39].

### Aspectos éticos

O estudo de coorte foi aprovado pela Comissão Nacional de Ética em Pesquisa (CONEP nº 719/2010) e pelo Comitê de Pesquisa da Escola Nacional de Saúde Pública da Fundação Oswaldo Cruz (CEP/ENSP nº 160/10). A Coorte Guarani foi autorizada pelas lideranças indígenas que assinaram o Termo de Consentimento Livre e Esclarecido, além do consentimento verbal individual das mães ou responsáveis ​​e da autorização da Fundação Nacional de Saúde do Brasil para o ingresso em territórios indígenas para fins de pesquisa científica. Este subprojeto da Coorte foi aprovado pelo Comitê de Ética em Pesquisa da Escola Nacional de Saúde Pública da Fundação Oswaldo Cruz (CEP/ENSP), protocolo número 1.821.137.

A autorização da Coorte de Nascimentos Guarani pelas comunidades indígenas teve início em 2013. A proposta previamente aprovada por outros fóruns éticos e de serviços de saúde foi apresentada na reunião do Conselho Distrital, fórum regional de controle social do SASI-SUS, igualmente composto por gestores de saúde, profissionais de saúde e usuários indígenas. Após essa aprovação, o coordenador da pesquisa visitou todas as aldeias do território durante um período de seis meses e apresentou os resultados de estudos anteriores às lideranças locais, membros da comunidade interessados ​​e profissionais de saúde locais, incluindo agentes comunitários indígenas de saúde, para solicitar autorização. para a nova pesquisa. O Termo de Consentimento Livre e Esclarecido Coletivo foi redigido em português, que é razoavelmente bem compreendido e falado pela maioria dos guaranis, embora os agentes indígenas de saúde e outros membros mais jovens das comunidades atuem sempre como mediadores de comunicação nas aldeias. Esta apresentação foi pautada pelo diálogo e os participantes tiveram a oportunidade de colocar questões e sugestões. Das 83 aldeias, apenas uma não tinha interesse em participar e 63 tinham condições adequadas para realizar a pesquisa.

A equipe de pesquisa realizou, então, sete workshops regionais e um workshop centralizado para capacitação técnica de equipes de saúde indígena na estratégia da OMS de Atenção Integrada às Doenças Prevalentes na Infância-AIDPI e nos procedimentos de vigilância e coleta de dados. O estudo desta coorte foi então iniciado em junho de 2014. O recrutamento de cada recém-nascido no período de 2 anos foi feito pela equipe de saúde local com a participação de agentes indígenas de saúde, que foram treinados para explicar a pesquisa e obter informações individuais verbais consentimento, conforme previsto no protocolo de pesquisa e aprovado pelo CEP/ENSP e pela CONEP, com base nas situações previstas na legislação nacional de pesquisa com povos indígenas. A recusa de consentimento ou posterior retirada desse consentimento foi possível a qualquer momento sem penalidade. Acreditamos que o projeto de pesquisa ajudou os pais a aprenderem a identificar eles próprios os sinais da gravidade da doença em seus filhos. Embora a iniciativa tenha se constituído em pesquisa científica, ela, no entanto, desempenhou um papel na atuação das equipes de saúde indígena, contribuindo para a capacitação técnica, a vigilância ativa e a identificação e manejo oportunos das doenças e agravos de alta prevalência na população infantil.

## Resultados

Houve 435 nascimentos elegíveis na área de estudo, e 74 (17,0%) destes não foram recrutados. Os sujeitos não recrutados foram: duas recusas, um óbito neonatal, três migrações fora da área de estudo logo após o nascimento e 68 falhas no recrutamento por lacunas na vigilância da Equipe Multidisciplinar de Saúde Indígena (EMSI). A Tabela 1 mostra uma comparação de características entre crianças recrutadas e não recrutadas.

**Table 1:**
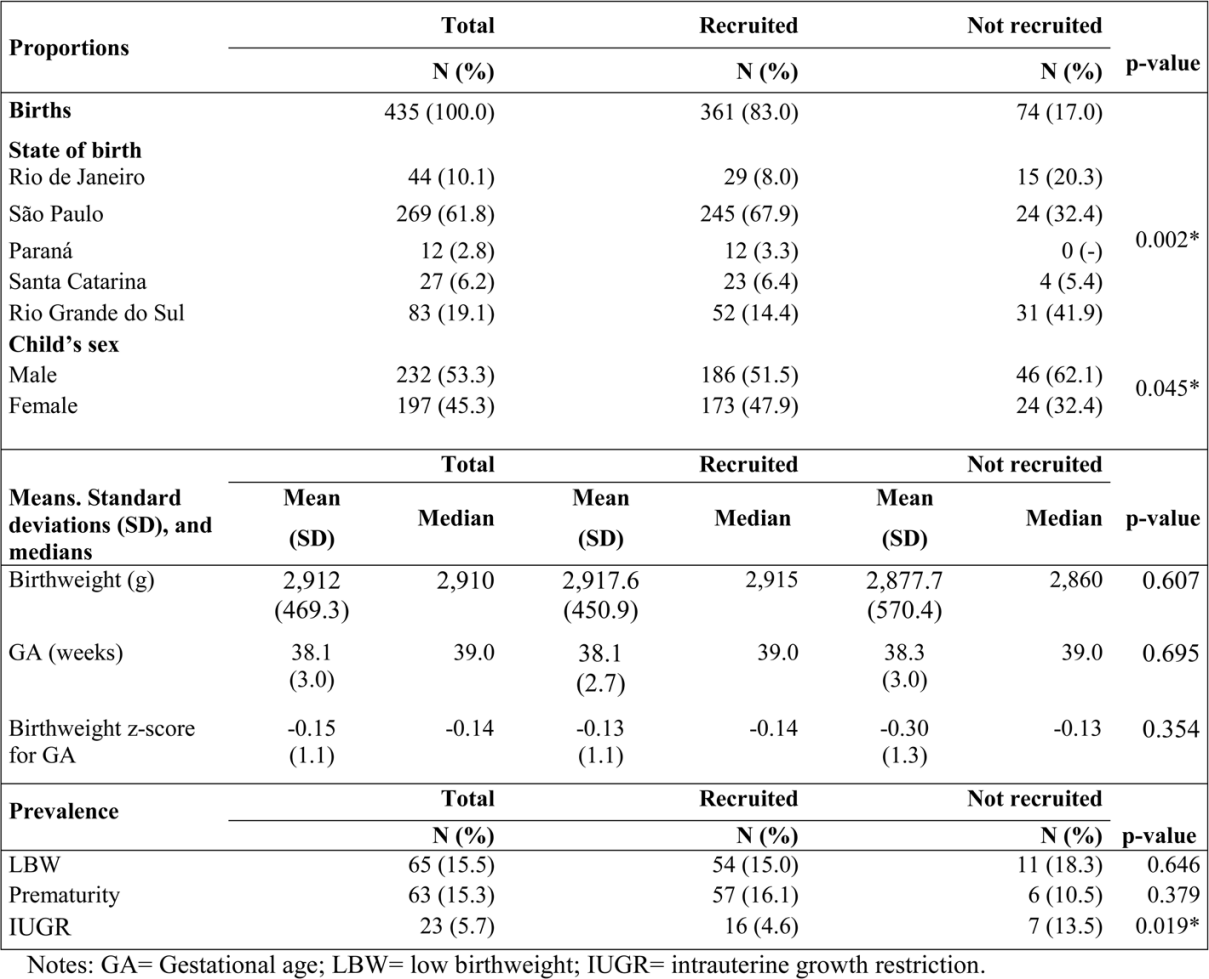
Análise comparativa das características selecionadas em crianças recrutadas e não recrutadas. Linha de Base da Coorte de Nascimentos Guarani, Sul e Sudeste do Brasil, 2014 - 2016.

Houve maiores proporções de não recrutamento entre os meninos e entre os nascimentos nos estados do Rio de Janeiro e Rio Grande do Sul (Quadro 1). Não foram observadas diferenças estatisticamente significativas no peso médio ao nascer, na idade gestacional ao nascer e no escore Z do peso ao nascer para a idade gestacional entre crianças recrutadas e não recrutadas. Tendo em vista o nível reduzido de dados faltantes, realizamos uma análise completa do caso.

As taxas gerais de prevalência de BPN, prematuridade e RCIU foram de 15,5, 15,3 e 5,7%, respectivamente. Não houve diferença estatisticamente significativa nas taxas de prevalência de BPN e prematuridade entre crianças recrutadas e não recrutadas. No entanto, a prevalência de RCIU foi significativamente menor em crianças recrutadas. Em mais de dois terços dos casos (69,8%), a prematuridade foi tardia (34 a 36 semanas). Quase metade (48,2%) dos bebês BPN nasceram prematuramente e um quarto teve RCIU.

A análise descritiva da linha de base da coorte e investigação dos determinantes dos desfechos a partir deste momento, bem como os resultados apresentados nas Tabelas 2, 3, 4 e 5, referem-se apenas aos 361 partos recrutados.

**Tabela 2:** Número e proporção de nascimentos e taxas de prevalência de BPN, prematuridade e RCIU segundo categorias de variáveis ​​exploratórias. Linha de base da Coorte de Nascimentos Guarani, Sul e Sudeste do Brasil, 2014-2016

Nível hierárquico, dimensão e variável	Nascimentos N (%)		Prevalência de BPN N (%)		Prevalência de prematuridade N (%)		Prevalência IUGR N (%)	
**Nível 1**								
**Características socioeconômicas**								
Renda per capita regular (U $ )								
Pobreza extrema	241	66,8	38	15,8	40	16,9	12	5,2
Pobreza	120	33,2	16	13,4	17	14,3	4	3,4
Acima da linha da pobreza	0	-	0	-	0	-	0	-
**Características domésticas**								
Principal fonte de energia para cozinhar								
Fogão a gás	178	49,3	20	11,3	17	9,8	9	5,2
Fogão à lenha	44	12,2	9	20,9	13	29,5	2	4,9
Fogueira	116	32,1	20	17,2	25	21,7	5	4,3
Carcaça de tijolo e argamassa								
Não	302	83,7	51	16,9	47	15,8	16	5,4
sim	59	16,3	3	5,2	10	17,5	0	
Cobertura adicional nas paredes e teto								
Não	149	41,3	17	11,5	29	19,9	6	4,3
sim	212	58,7	37	17,5	28	13,4	10	4,8
**Características maternas**								
Idade materna (anos)								
<20	133	36,8	29	21,8	25	19,1	9	6,9
20–34	196	54,3	21	10,8	25	13,0	6	3,2
≥ 35	32	8,9	4	12,5	7	21,9	1	3,1
Altura materna (cm)								
> 150	116	32,1	13	11,2	16	14,0	5	4,4
≤ 150	234	64,8	39	16,8	39	16,8	10	4,4
Escolaridade materna								
Secundário ou universidade	40	11,1	6	15.0	4	10,0	2	5.0
Primário	188	52,1	28	15.0	25	13,6	11	6,1
Sem escolaridade formal	107	29,6	14	13,2	23	21,5	2	1,9
Estado civil								
Com marido / companheiro	279	77,3	40	14,4	43	15,6	10	3,7
Sem marido / companheiro	82	22,7	14	17,3	14	17,7	6	7,7
**Nível 2**								
**Variáveis ​​reprodutivas**								
Paridade								
Multíparas	276	76,5	34	12,4	39	14,3	7	2,6
Primípara	84	23,3	20	23,8	18	22,0	9	11,1
Natimortos ou abortos								
Não	65	18,0	8	12,3	8	12,3	1	1,6
sim	290	80,3	45	15,6	48	16,8	14	5.0
**Qualidade do atendimento pré-natal**								
Indicador limitado								
Adequado	207	57,3	26	12,7	25	12,2	10	5.0
Inadequada	153	42,4	28	18,3	32	21,5	6	4,1
Indicador expandido								
Adequado	184	51.0	24	13.2	22	12.0	9	5.0
Inadequate	176	48.8	30	17.0	34	19.9	7	4.1
**Level 3**								
**Maternal morbidity**								
Hypertension								
No	345	95.6	50	14.6	55	16.1	14	4.2
Yes	9	2.5	3	33.3	1	11.1	1	11.1
Urinary infection								
No	260	72.0	37	14.3	43	16.7	9	3.6
Yes	94	26.0	16	17.0	13	14.0	6	6.5
**Maternal nutrition**								
Chronic anemia before pregnancy								
No	344	95.3	48	14.0	54	15.9	13	3.9
Yes	10	2.8	5	50.0	2	20.0	2	20.0
Pre gestational nutritional status								
Underweight	9	2.5	2	22.2	2	22.2	1	11.1
Adequate weight	211	58.4	37	17.6	35	16.7	8	3.9
Excess weight	112	31.0	10	9.0	14	12.6	4	3.7
Gestational weight gain								
Insufficient	205	56.8	35	17.2	32	15.6	6	3.0
Adequate	69	19.1	7	10.3	10	14.5	5	7.4
Excessive	47	13.0	6	12.8	7	14.9	2	4.3
**Maternal habits**								
Smoking in pregnancy								
No	340	94.2	52	15.4	56	16.8	15	4.6
Yes	20	5.5	2	10.0	1	5.0	1	5.0
Alcohol use in pregnancy								
No	336	93.1	48	14.4	52	15.7	15	4.6
Yes	22	6.1	6	27.3	5	23.8	1	4.8
**Type of deliveries (hospital only)**								
Vaginal	207	57.3	35	17.0	33	16.2	10	5.0
Cesarean	43	11.9	5	11.6	3	7.1	1	2.3
**Child’s sex**								
Female	173	47.9	31	17.9	26	15.3	–	–
Male	186	51.5	22	12.0	31	16.8

**Table 3:** Crude associations between socioeconomic, household, and maternal characteristics (Hierarchical Level 1) andLBW, prematurity, andIUGR. Baseline of Guarani Birth Cohort, South and Southeast Brazil, 2014–2016

Hierarchical level, dimension, and variable	LBW		Prematurity		IUGR	
	OR (95%CI)	***p***-value	OR (95%CI)	***p***-value	OR (95%CI)	***p***-value
**Level 1**						
**Socioeconomic characteristics**						
Regular per capita income (U$)						
Extreme Poverty	1.21 (0.64–2.27)	0.552	1.22 (0.66–2.27)	0.519	1.55 (0.49–4.93)	0.454
Poverty	1.00		1.00		1.00	
**Household characteristics**						
Principal energy source for cooking						
Gas stove	1.00		1.00		1.00	
Wood burning stove	2.08 (0.87–4.96)	0.099*	3.87 (1.71–8.78)	0.001*	0.93 (0.19–4.5)	0.933
Bonfire	1.64 (0.84–3.20)	0.150*	2.57 (1.31–5.01)	0.006*	0.83 (0.27–2.54)	0.742
Brick and mortar housing						
No	1.00		1.00		1.00	
Yes	0.27 (0.08–0.89)	0.031*	1.14 (0.54–2.41)	0.739	–	
Additional covering on walls or ceiling						
No	1.00		1.00		1.00	
Yes	1.64 (0.88–3.04)	0.117*	0.62 (0.35–1.1)	0.104*	1.13 (0.4–3.18)	0.816
**Maternal characteristics**						
Maternal age (years)						
< 20	2.3(1.25–4.24)	0.008*	1.58 (0.86–2.89)	0.141*	2.26 (0.78–6.5)	0.132*
20–34	1.00		1.00		1.00	
≥ 35	1.18 (0.38–3.68)	0.780	1.87 (0.73–4.78)	0.191*	0.98 (0.11–8.41)	0.984
Maternal height (cm)						
> 150	1.00		1.00		1.00	
≤ 150	1.6 (0.82–3.13)	0.170*	1.24 (0.66–2.33)	0.507	1.01 (0.34–3.03)	0.987
Maternal schooling						
Secondary or university	1.16 (0.41–3.26)	0.779	0.41 (0.13–1.26)	0.118*	2.66 (0.36–19.54)	0.337
Primary	1.16 (0.58–2.31)	0.679	0.57 (0.31–1.07)	0.082*	3.27 (0.71–15.04)	0.128*
No formal schooling	1.00		1.00		1.00	
Marital status						
With husband/partner	1.00		1.00		1.00	
Without husband/partner	1.24 (0.64–2.42)	0.522	1.17 (0.6–2.26)	0.648	2.18 (0.77–6.21)	0.143*

*LBW* Low birthweight, *IUGR* Intrauterine growth restriction

*Categories of independent variables associated with the outcome, with levelof significance *p* < 0.20, indicating that the variable was included in the multivariate analysis at its hierarchical level

**Table 4:** Crude associations between reproductive variables, quality and access to prenatal care, maternal morbidity, and habits in the pregnancy, place and type of delivery, and child’s sex (hierarchical levels 2 and 3) and LBW, prematurity, and IUGR. Baseline of the Guarani Indigenous Cohort, South and Southeast Brazil, 2014–2016

Hierarchical level, dimension, and variable	LBW		Prematurity		IUGR	
	OR (95%CI)	***p***-value	OR (95%CI)	***p***-value	OR (95%CI)	***p***-value
**Level 2**^**a**^						
**Reproductive variables**						
Parity						
Multiparous	1.00		1.00		1.00	
Primiparous	1.58 (0.77–3.24)	0.216	1.73 (0.89–3.33)	0.104*	4.66 (1.68–12.95)	0.003*
Abortions and stillbirths						
No	1.00		1.00		1.00	
Yes	1.14 (0.5–2.64)	0.749	1.48 (0.65–3.38)	0.356	3.3 (0.43–25.57)	0.252
**Quality of prenatal care**						
Limited indicator						
Adequate	1.00		1.00		1.00	
Inadequate	1.69 (0.92–3.09)	0.091*	1.71 (0.94–3.11)	0.076*	0.82 (0.29–2.3)	0.702
Expanded indicator						
Adequate	1.00		1.00		1.00	
Inadequate	1.47 (0.8–2.69)	0.215	1.56 (0.85–2.85)	0.152*	0.82 (0.3–2.26)	0.702
**Level 3**^**b**^						
**Maternal morbidity**						
Hypertension						
No	1.00		1.00		1.00	
Yes	3.84 (0.88–16.74)	0.074*	0.80 (0.09–6.84)	0.839	5.21 (0.56–48.48)	0.147*
Urinary infection in pregnancy						
No	1.00		1.00		1.00	
Yes	1.18 (0.61–2.29)	0.624	1.23 (0.60–2.51)	0.575	0.53 (0.18–1.57)	0.254
**Maternal nutritional status**						
Chronic anemia before pregnancy						
No	1.00		1.00		1.00	
Yes	6.41 (1.7–24.16)	0.006*	1.56 (0.30–8.03)	0.598	7.21 (1.29–40.38)	0.025*
Pregestational nutritional status						
Underweight	1.32 (0.24–7.12)	0.753	2.53 (0.45–14.15)	0.289	2.22 (0.24–4.12)	0.486
Adequate	1.00		1.00		1.00	
Excess weight	0.54 (0.24–1.18)	0.12*	0.80(0.39–1.61)	0.524	1.17 (0.33–4.12)	0.805
Gestational weight gain						
Insufficient	1.71 (0.71–4.12)	0.228	0.85 (0.38–1.91)	0.691	0.38 (0.11–1.3)	0.123*
Adequate	1.00		1.00		1.00	
Excessive	1.37 (0.42–4.47)	0.599	0.99 (0.33–2.91)	0.979	0.53 (0.1–2.89)	0.123*
**Maternal habits in pregnancy**						
Smoking						
No	1.00		1.00		1.00	
Yes	1.35 (0.3–6.13)	0.701	2.72 (0.35–21.29)	0.34	0.75 (0.09–6.23)	0.791
Alcohol use						
No	1.00		1.00		1.00	
Yes	2.13 (0.77–5.87)	0.145*	1.83 (0.33–10.12)	0.489	0.77 (0.09–6.34)	0.805
**Type of delivery (hospital)**						
Vaginal	1.00		1.00		1.00	
Cesarean	0.76 (0.27–2.12)	0.597	1.66 (0.29–9.57)	0.569	0.39 (0.05–3.22)	0.385
**Child’s sex**						
Female	1.52 (0.83–2.78)	0.174*	0.80 (0.44–1.45)	0.461	NA	NA
Male	1.00		1.00			

*LBW* Low birthweight, *IUGR* Intrauterine growth restriction, *NA* Not applicable

*Categories of independent variables associated with the outcome, with level of significance *p* < 0.20, indicating that the variable was included in the multivariate analysis at its hierarchical level

^a^OR of level 2 variables in the hierarchical model were adjusted for the variables that remained in level 1

^b^OR of level3 variables in the hierarchical model were adjusted for the variables that remained in levels 1 and 2

**Table 5:** Final hierarchical multivariate logistic regression model for risk factors for LBW, prematurity, and IUGR. Baseline of the Guarani Birth Cohort, South and Southeast Brazil, 2014–2016

Level	Domains	Variables	OR^**a**^ (95%CI)	***p***-value
	**Low birthweight**			
**1**	**Household characteristics**	Brick and mortar housing		
		No	1.00	
		Yes	0.25 (0.07–0.84)	0.025
**1**	**Maternal characteristics**	Maternal age (years)		
		< 20	2.39 (1.29–4.44)	0.006
		20 to 34	1.00	
		≥ 35	1.15 (0.36–3.61)	0.815
**3**	**Maternal nutrition**	Chronic anemia before pregnancy		
		No	1.00	
		Yes	6.41 (1.70–24.16)	0.006
	**Prematurity**			
**1**	**Household characteristics**	Principal energy source for cooking		
		Gas stove	1.00	
		Wood burning stove	3.87 (1.71–8.78)	0.001
		Bonfire	2.57 (1.31–5.01)	0.006
	**Intrauterine growth restriction**			
**2**	**Reproductive variables**	Parity		
		Multiparous	1.00	
		Primiparous	4.66 (1.68–12.95)	0.003
**3**	**Maternal nutrition**	Chronic anemia before pregnancy		
		No	1.00	
		Yes	7.21 (1.29–40.38)	0.025

Note: ^a^adjusted OR Each variable’s effect on the outcome was adjusted for the other variables at the same hierarchical level that remained with *p* < 0.05 at the end of the multivariate analysis of the respective level and for the variables that remained from the previous levels. The odds ratios refer to the sizes of adjusted associations reached at entry level of each of these variables in the hierarchical model

All of the Guarani children recruited for the study lived below the poverty line, and two-thirds (66.3%) were below the cutoff for extreme poverty. More than 30% of the children lived in households that used bonfires as their principal energy source for cooking, 83.7% lived in houses built of logs, wood, straw, mud, or other similar materials, and more than half of these households (58.7%) used some additional material such as tarpaulins, plastic sheeting or blankets to cover the ceiling or walls (Table 2).

The proportion of mothers under 20 years of age was 36.8%, with mean maternal age of 23.9 years (SD: 7.6) and median of 22 years; 64.8% of the mothers were ≤ 150 cm tall, with mean and median maternal height of 148.5 cm (SD: 5.6) and 148.0 cm, respectively. Almost two thirds (63.2%) of the mothers had some formal education, 77.3% had a husband or partner, the majority were multiparous (76.5%) and had an obstetric history of stillbirths or abortions (80.3%) (Table 2).

Slightly more than 50% of the mothers of recruited children were classified as having adequate prenatal care, according to either of the indicators used. In 2.5% of the pregnancies there was pregestational and/or gestational hypertension. Urinary infection was diagnosed at some moment in 26.0% of the pregnancies; 2.8% of the mothers presented chronic anemia before pregnancy and 58.4% had adequate nutritional status when they became pregnant. However, 56.8% showed insufficient weight gain during the pregnancy, and 13.0% showed excessive weight gain. Prevalence rates for smoking and alcohol use during the pregnancy were 5.5 and 6.1%, respectively. Hospital deliveries represent 70.9% of all Guarani deliveries and the cesarean section prevalence rate was 11.9% in the hospital deliveries (Table 2).

Five variables from hierarchical level 1 showed crude significant association with LBW at *p* < 0.20 (Table 3). Use of bonfires (OR: 1.64, CI95%: 0.84–3.20) or wood-burning stove (OR: 2.08, 0.87–4.96) as the main energy source for cooking, use of additional covering on walls or ceiling (OR: 1.64, CI95%: 0.88–3.04), and low maternal height (OR: 1.6, CI95%: 0.82–3.13) lost statistical significance in the multivariate analysis. The only variables that remained in the final model were brick and mortar housing (OR: 0.27, CI95%: 0.08–0.89) and maternal age (< 20 - OR: 2.3, CI95%: 1.25–4.24; ≥ 35 – OR: 1.18, CI95%: 0.38–3.68), with significance at *p* < 0.05.

After adjusting for the variables kept in level 1, quality of prenatal care (limited indicator) (OR: 1.69, CI95%: 0.92–3.09) was the only variable in level 2 that was associated with LBW. As its significance was above *p* < 0.05, we left it out of the final model (Table 4). The variables hypertension (OR: 3.84, CI95%: 0.88–16.74), chronic anemia before pregnancy (OR: 6.41, CI95%: 1.7–24.16), pregestational nutritional excess weight (OR: 0.54; CI95%: 0.24–1.118), alcohol use in pregnancy (OR: 2.13, CI95%: 0.77–5.87), and child’s female sex (OR: 1.52, CI95%: 0.83–2.78) showed significance at *p* < 0.20 and were included in the multivariate analysis in level 3, together with the variables kept in level 1 (Table 4).

At the end of the multivariate analysis in level 3, only chronic anemia before pregnancy remained associated with LBW, with significance at *p* < 0.05. In the final model, LBW was significantly associated with maternal age (< 20 - OR: 2.39, CI95%: 1.29–4.44), brick and mortar housing (OR: 0.25, CI95%: 0.07–0.84) and chronic maternal anemia before pregnancy (OR: 6.41, CI95%: 1.70–24.16) (Table 5).

Four variables from hierarchical level 1 showed crude association with prematurity at *p* < 0.20 (Table 3). Use of additional materials to cover the walls or ceiling (OR: 0.62, CI95%: 0.35–1.1) and maternal schooling (secondary or university – OR: 0.41, CI95%: 0.13–1.26; primary – OR: 0.57, CI95%: 0.31–1.07) lost statistical significance in the multivariate analysis, and the variable kept in the final model for level 1 was the principal energy source for cooking (wood-burning stove – OR: 3.87, CI95%: 1.71–8.78; bonfire - OR: 2.57, CI95%: 1.31–5.01), with significance at *p* < 0.05. Parity and quality of prenatal care were included in the level 2 multivariate analysis, together with the variable kept at level 1, but none of them remained statistically significant in the adjusted analysis. In the final model, prematurity was significantly associated with the type of principal energy source for cooking (wood-burning stove - OR: 3.87, CI95%: 1.71–8.78; bonfire - OR: 2.57, CI95%: 1.31–5.01) (Table 5).

With IUGR as the outcome, maternal age (< 20 - OR: 2.26, CI95%: 0.78–6.5), schooling (primary - OR: 3.27, CI95%: 0.71–15.04), and marital status (without husband/partner - OR: 2.18, CI95%: 0.77–6.21) showed crude associations with IUGR, with significance at *p* < 0.20 (Table 3). No level 1 variables remained in the final model with significance at *p* < 0.05. In the level 2 crude analysis, primiparous (OR: 4.66, CI95%: 1.68–12.95) was the only variable significantly associated with IUGR, with significance at *p* < 0.05 (Table 4). The variables hypertension, chronic maternal anemia before pregnancy, and gestational weight gain were added to the multivariate analysis in level 3, but chronic maternal anemia before pregnancy (OR: 7.21, CI95%: 1.29–40.38) was the only variable that remained in the final model (*p* < 0.05). In the final model, IUGR showed a statistically significant association with primiparity (OR: 4.66, CI95%: 1.68–12.95) and chronic anemia before pregnancy (OR: 7.21, CI95%: 1.29–40.38) (Table 5).

## Discussion

The prevalence rates for LBW (15.0%) and prematurity (16.1%) were high in the Guarani population in South and Southeast Brazil and exceeded the corresponding prevalence rates in most indigenous and non-indigenous populations. However, prevalence of IUGR (4.6%) was similar to or lower than in other populations. These outcomes were associated with household conditions, lower maternal age, maternal nutritional status at the beginning of pregnancy, and obstetric causes.

Prevalence of LBW in the Guarani was almost double that of the corresponding prevalence rates in the non-indigenous Brazilian population (6.1–8.0%) [39], among indigenous peoples as a whole in Brazil (7.3%) [40], and in indigenous peoples in other regions of the world (6.1%) [41]. It was only exceeded by the reported prevalence rates among the Aboriginal peoples in remote areas of Australia (17.4%) [15]. Prevalence of prematurity in the Guarani was also higher than the corresponding rates in the non-indigenous Brazilian population (11.5%) [42] and in indigenous peoples in other regions of the world (10.8%) [41], and was only lower than the prevalence rates among the Aboriginal peoples of Australia (19.4%), which were the highest prevalence rates of prematurity reported in the literature [15]. Meanwhile, prevalence of IUGR in the Guarani was lower than the rates described in non-indigenous populations in Brazil that used the same reference curve as this study (9.3%) [43]. Prevalence was close to the rates reported in indigenous populations in different regions of the world (7.8%) [41], and lower than the prevalence rates in Aboriginal peoples in Australia (16.3%) [15]. However, the curves used to assess fetal growth in the latter two studies were different, which may limit direct comparisons of the results. No studies were found that describe the prevalence of prematurity or IUGR in indigenous peoples in Brazil.

Household per capita income, used as the socioeconomic indicator in our study, showed that all the liveborn Guarani infants lived in households below the poverty line, and that two-thirds were living in extreme poverty. This shows that the study population suffers unfavorable socioeconomic conditions when compared to the mean levels in the Brazilian population as a whole and among the non-indigenous population of the geographic regions of the study villages [35]. However, this indicator, household per capita income, was not associated with any of the three outcomes in the final model. This may be due to the relative socioeconomic homogeneity of the indigenous group when this dimension is measured by traditional indicators like per capita income. This indicator was used in order to allow comparisons with other studies and due to difficulties in building sensitive indicators for capturing intragroup socioeconomic differences [12]. However, the Guarani traditionally practice a barter system between family groups and rely on subsistence farming, the sale of traditional products and handicrafts, and donations [44]; the income indicator used here may thus not reflect their true socioeconomic situation.

Housing conditions have been identified in the literature as a factor associated independently with LBW and prematurity, reflecting socioeconomic conditions in urban populations in Brazil [45]. Living in brick and mortar housing was associated with 75% lower odds of LBW in the Guarani. Meanwhile, a study of indigenous people in Ecuador showed that less urbanized dwellings were more adequate in different aspects of their occupants’ health when compared to more urbanized dwellings [46].

Although the Guarani villages showed some internal homogeneity in their housing standards, there is considerable heterogeneity between villages. The housing ranges from makeshift shacks, log-and-straw houses, packed-earth walls, and reed houses to brick and mortar houses, sometimes in different combinations of these types. Such housing standards are largely determined by characteristics of the respective villages, such as territorial extensions of (and proximity to) cities, availability of building materials in the local ecosystem, or access to targeted public housing policies [47]. Thus, housing standards in the Guarani are not a good socioeconomic indicator, but rather act as a proxy for exposure to environmental risks such as indoor pollution and crowding [23]. The interpretation of this association should thus consider the possibility that this indicator partly expresses the effect of other unmeasured socioeconomic variables or exposure to indoor pollutants [44]. There is no doubt that public housing measures are necessary for indigenous peoples, but they need to take into account more specific studies on the standards that can reduce environmental and health risks, necessarily considering the wishes and knowledge of the people.

The use of biomass as an energy source for cooking and heating is common among indigenous peoples and rural populations in Brazil and elsewhere in the world. The use of wood-burning stoves or bonfires in Guarani households was associated with an almost four-fold increase in the odds of prematurity, compared to children of mothers with gas stoves. Exposure to indoor pollution resulting from burning solid fuels has been associated with increased risk of adverse pregnancy outcomes such as LBW and prematurity [48]. The biological mechanisms involved in the association with fetal development appear to be related to placental alterations and reduced maternal lung function [48]. Smoke from burning biomass contains various pollutants that can lead to different pulmonary and placental inflammatory responses due to oxidative stress, influencing endothelial functions and triggering hemodynamic responses that limit fetal growth [48, 49].

Studies have reported higher prevalence of LBW and prematurity at the extremes of women’s childbearing years [16, 50, 51]. These effects are assumed to be related to intrinsic age-related biological factors such as physiological immaturity for pregnancy in younger women, especially under 15 years of age, and higher prevalence of pregnancy-induced hypertension and hemorrhage in older women [52, 53]. Our study corroborated some of these results and found that LBW was associated with maternal age of less than 20 years. Oster and Toth [16], in a study of First Nations in Canada, reported a nearly twofold risk of LBW in mothers over 35 years of age. Kildea et al. [15] identified maternal age of less than 20 years as a risk factor for prematurity in the Australian Aboriginal population. Coversely, Heaman et al. [17] found a protective effect of maternal age of less than 19 years on prematurity in First Nations in Canada, and Oster and Toth [16] reported a protective effect of maternal age of less than or equal to 17 years on LBW, also in First Nations of Canada. Pregnancy in younger Guarani women does not appear to be a marker of social disadvantage since pregnancy in this age bracket is an event expected by the community [54]. We may thus assume that biological mechanisms are involved in the determination of LBW among the Guarani, indicating the importance of access to prenatal care, above all in this age bracket.

The odds of IUGR in primiparous Guarani women were 4.6 times those of multiparous mothers. This finding corroborates the findings from previous studies in both indigenous [15] and non-indigenous populations [50]. According to Bernabé et al. [50], the vascular maturation of uterine structures occurs in the first pregnancy, making the structures more sensitive to gestational stimuli. In subsequent pregnancies, the maturity of the reproductive structure allows more appropriate placental development and thus better fetal nutrition [50].

Anemia remains an important public health problem in indigenous women in Brazil, with prevalence rates ranging from 16.1% in non-pregnant women to 81.8% in pregnant women [55]. The First National Health and Nutrition Survey of Indigenous Peoples in Brazil confirmed for the first time on a national and regional scale, the high prevalence of anemia in indigenous women of childbearing-age (33.0% in the country as a whole and 30.8% in the South and Southeast, where the Guarani villages are located [56]. Anemia is known to begin or to be exacerbated in pregnancy due to the increased plasma volume and resulting plasma dilution [57]. Prevalence of chronic anemia before pregnancy in our study was considerably lower than these values, and may have been underestimated. Nevertheless, it was possible to detect a significant association between anemia and LBW and IUGR, as found in other studies [50, 57, 58]. Low hemoglobin levels favor alteration in placental angiogenesis, limiting the availability of oxygen to the fetus and thus causing potential IUGR and LBW [59]. This result emphasizes the importance of access to prenatal care and supplementation with ferrous sulfate and treatment of anemia diagnosed before and during pregnancy.

Despite the expansion of access and almost universal coverage of prenatal care in Brazil in recent decades, regional and social inequalities still exist in access to adequate care, which can be illustrated by differences in the trimester of prenatal care initiation, the number of consultations and the availability of testing for syphilis and HIV [36]. For indigenous peoples, there has also been a dramatic expansion of access to prenatal care in the last two decades, based on the implementation of the SASI-SUS, when primary health care started to be provided at village level. However, the quality of such prenatal care remains far short of the target and lower than in the non-indigenous Brazilian population, with later initiation, fewer consultations, poor clinical and laboratory monitoring and worse perinatal outcomes [26]. Better quality of prenatal care leads to better adoption of preventive measures and timely access to effective interventions for the control of biological and environmental risk factors associated with prematurity [36, 42]. This result reinforces the importance of expanding primary care in the villages and encouraging adherence to prenatal care.

Prevalence of induced labor in Brazil is one of the highest in the world, and Brazil thus also has one of the highest cesarean rates [42]. Induced labor has been identified as an iatrogenic risk in the determination of prematurity and LBW in Brazil. For the Guarani population in this study, the cesarean rate was low compared to overall rates in Brazil, and it was within the limits recommended by the World Health Organization (WHO) [60]. Thus, in the case of the Guarani population, although prematurity consisted predominantly of late premature infants (34 to 36 weeks), no association was seen between cesarean delivery and prematurity. While on the one hand this is a good indicator of the use of primary and hospital care by the indigenous population, on the other it indicates the need to both expand access to prenatal care and to improve the quality of such care. In addition, unexpectedly, Guarani children born by cesarean delivery showed significantly higher mean weight (3095.0 g) than children born by vaginal delivery (2890.5) (*p* < 0.026), which emphasizes adverse environmental conditions, maternal infections, and poor quality of prenatal care as determinants of prematurity in the Guarani.

Some limitations should be addressed in the interpretation of this study’s findings. The first point is that the outcomes are prevalent by their nature, since liveborn infants are survivors of a conception cohort, while abortions and stillbirths may have occurred in this population. This is a common and almost insurmountable characteristic of studies on LBW, prematurity, and IUGR, and we can assume that the results are affected to some degree by survival bias. Studies indicate the possibility of birthweight measurement bias, especially in homebirths and populations with low socioeconomic status. Our study did not detect statistically significant differences in the prevalence rates of LBW (*p* < 0.50) or mean birthweight (*p* < 0.85) when comparing children born in the villages with those born in hospital. We also identified the occurrence of preferred digits (0 and 5) in the birthweight variable, but this occurred in both homebirths and hospital births and in all the ranges of weight measurement. Thus, possible errors in LBW classification appear to have occurred by chance.

The low prevalence of IUGR in comparison to LBW and prematurity could suggest that measurement bias has occurred. Clearly, gestational age could have been underestimated by recall bias related to the date of the last menstruation, or loss of precision of ultrasound at advanced gestational age. Weight could also be affected by measurement bias, as mentioned previously. On the other hand, both LBW and prematurity would be underestimated by these same potential biases, and this does not seem to have occurred. Since the prevalence of IUGR was significantly higher among non-recruited children, we suggest selection bias as the most likely explanation. In addition, some studies indicate that the INTERGROWTH 21st curve tends to underestimate the frequency of small for gestational age newborns, a proxy for IUGR [43]. Despite this, our study detected associations with anemia before pregnancy and primiparity. We believe that if the IUGR prevalence is underestimated, the real magnitude of the associations would be even greater, and additional unconfirmed associations could have been found.

Our results show biological plausibility and strong consistency with the literature. The analyses were performed at the baseline of the first indigenous birth cohort in Brazil and the recruitment level was high, without statistical differences in the prevalence rates of LBW and prematurity or in birthweight, gestational age at birth, and birthweight z-score for gestational age between recruited and unrecruited children, which reinforces the robustness of the study.

As mentioned previously, Brazil has one of the world’s most socially diverse indigenous populations. The indigenous category encompasses extensive differences in culture, geographical and territorial occupation, degrees of contact with the surrounding society, exposure to environmental and climatic conditions, health standards and access to natural resources, goods and services, and public policies, such as health. Although this diversity imposes a certain limitation on the generalization of Guarani results to all indigenous peoples in the country, our study identified a common pattern marked by the social and health disadvantage of the indigenous people compared to their counterparts. Thus, these results can serve as a topic for debate about inequities in health and expanding access to public policies to guarantee the constitutional rights of indigenous peoples in Brazil.

## Conclusions

As mentioned previously, the identification of populations at greater risk of LBW and that face barriers in access to health and nutrition policies is a global health priority. This study revealed high prevalence rates for LBW and prematurity in the Guarani indigenous population, higher than reported in most studies in indigenous and non-indigenous peoples in Brazil and elsewhere. The target outcomes were associated with environmental determinants such as quality of the household environment, maternal nutritional and health status before and during pregnancy, obstetric history, and access to and use of health services, most of which are modifiable through interventions by the health sector and by inter-sector policies on food security, housing, and the environment. Emphasis should be given to strengthening the Indigenous Healthcare Subsystem, allowing improved access to primary healthcare by indigenous peoples and the development of culturally sensitive health practices.

Interventions in these fields may result not only directly in the reduction of prevalence rates for LBW, prematurity, and IUGR in the population, but also indirectly in the reduction of the high burden of morbidity and mortality from infectious diseases, especially acute respiratory infections, and malnutrition in childhood. They could also minimize delays in cognitive development, preventing chronic noncommunicable diseases in adulthood, and interrupting the vicious circle of poverty and social exclusion that historically affects indigenous peoples in Brazil.

## Supplementary Information

**Additional file 1 Supplementary file 1.** The Guarani Birth Cohort - Perinatal Questionnaire Original Version (portuguese). The Perinatal questionnaire comprised 102 questions and some dependent subquestions, that were organized in blocks to be completed during the interview with the mother (33 items). Some information was extracted from secondary registries (69 items), to avoid unnecessarily long interviews for the participant. The total time for completing the questionnaire was about an hour, including a 30-min interview. The questionnaire was designed in digital format, operated on a handheld personal digital assistant (PDA), with subsequent online data transmission to the study coordinators. The perinatal questionnaire was designed by the research team and was based on questionnaires from the National Census, the First National Survey of Indigenous People’s Health and Nutrition in Brazil, a population-based case-control study in the Guarani population, and other Brazilian reference studies on infant-maternal health, such as the four Pelotas Birth Cohorts (1982, 1993, 2004 and 2015) and the Birth in Brazil Study. We also added questions based on the forms routinely used in health services for prenatal care, the hospital discharge summary after birth, the health card of the child, and the birth certificate.

**Additional file 2 Supplementary file 2.** The Guarani Birth Cohort - Perinatal Questionnaire English version (translated). The Perinatal questionnaire comprised 102 questions and some dependent subquestions, that were organized in blocks to be completed during the interview with the mother (33 items). Some information was extracted from secondary registries (69 items), to avoid unnecessarily long interviews for the participant. The total time for completing the questionnaire was about an hour, including a 30-min interview. The questionnaire was designed in digital format, operated on a handheld personal digital assistant (PDA), with subsequent online data transmission to the study coordinators. The perinatal questionnaire was designed by the research team and was based on questionnaires from the National Census, the First National Survey of Indigenous People’s Health and Nutrition in Brazil, a population-based case-control study in the Guarani population, and other Brazilian reference studies on infant-maternal health, such as the four Pelotas Birth Cohorts (1982, 1993, 2004 and 2015) and the Birth in Brazil Study. We also added questions based on the forms routinely used in health services for prenatal care, the hospital discharge summary after birth, the health card of the child, and the birth certificate.

## Data Availability

The dataset generated in the Guarani birth cohort is not yet publicly available. Due to the relatively small size of the study population, the dataset may not be adequately anonymized to permit open access and protect the participants’ identities. Proposals for access to data will be considered subject to ethical and legal restrictions, the terms of the original informed consent agreement with participant community, and Guarani community protocols for authorizing studies. Data requests may be sent to Dr. Ricardo Ventura Santos, leader of Health, Epidemiology and Anthropology Research Group, Departamento de Endemias Samuel Pessoa, Escola Nacional de Saúde Pública, Fundação Oswaldo Cruz, located at Rua Leopoldo Bulhões 1480, Rio de Janeiro, RJ 21041–210, Brazil (http://www.ensp.fiocruz.br/portal-ensp/departamento/densp/grupos-de-pesquisa). Phone: + 55 (21) 2598–2654. Email: santos@ensp.fiocruz.br.

## Abreviações

95% CI: Intervalos de confiança de 95%
ARIs: Infecções respiratórias agudas
DLM: Data da última menstruação
EMSI: Equipes Multidisciplinares de Saúde Indígena
LBW: Baixo peso de nascimento
INTERGROWTH-21st: Consórcio Internacional de Crescimento Fetal e Recém-nascido para o Século XXI
IOM: Instituto de Medicina
IUGR: Restrição de crescimento intrauterino
OU: Odds ratios
PDA: Assistentes digitais pessoais
SASI-SUS: Subsistema de Saúde Indígena
SUS: Sistema Único de Saúde Nacional
SD: Desvio padrão
WHO: Organização Mundial de Saúde
