# Correction to: Low birthweight, prematurity, and intrauterine growth restriction: results from the baseline data of the first indigenous birth cohort in Brazil (Guarani Birth Cohort)

**DOI:** 10.1186/s12884-020-03491-w

**Published:** 2020-12-30

**Authors:** Carla Tatiana Garcia Barreto, Felipe Guimarães Tavares, Mariza Theme-Filha, Yasmin Nascimento Farias, Lídia de Nazaré Pantoja, Andrey Moreira Cardoso

**Affiliations:** 1 Universidade do Estado do Rio de Janeiro (UERJ), Av. Marechal Rondon, 381. São Francisco Xavier, Rio de Janeiro, RJ CEP: 20950-000 Brasil; 2 Escola Nacional de Saúde Pública, Fundação Oswaldo Cruz, Rio de Janeiro, RJ Brasil; 3 Escola de Enfermagem Aurora de Afonso Costa. Faculdade de Enfermagem, Universidade Federal Fluminense, Niterói, RJ Brasil

**Correction to: BMC Pregnancy Childbirth 20, 748 (2020)**

**https://doi.org/10.1186/s12884-020-03396-8**

An error occurred during the publication of the original article [1] which led to the text being incorrectly converted into Portuguese.

The Publisher apologizes for this error and any confusion caused.

The original article has been corrected.
