# Supplementary material for: Baixo peso ao nascer, prematuridade e restrição de crescimento intra-uterino: resultados dos dados de base da primeira coorte de nascimentos indígenas no Brasil (coorte de nascimentos Guarani)
Source: BMC Pregnancy Childbirth. 2020 Dec 2;20:748. doi: 10.1186/s12884-020-03396-8 (PMC7709282; doi:10.1186/s12884-020-03396-8)
Supplement: Supplementary file 1 — Additional file 1 Supplementary file 1. The Guarani Birth Cohort - Perinatal Questionnaire Original Version (portuguese). The Perinatal questionnaire comprised 102 questions and some dependent subquestions, that were organized in blocks to be completed during the interview with the mother (33 items). Some information was extracted from secondary registries (69 items), to avoid unnecessarily long interviews for the participant. The total time for completing the questionnaire was about an hour, including a 30-min interview. The questionnaire was designed in digital format, operated on a handheld personal digital assistant (PDA), with subsequent online data transmission to the study coordinators. The perinatal questionnaire was designed by the research team and was based on questionnaires from the National Census, the First National Survey of Indigenous People’s Health and Nutrition in Brazil, a population-based case-control study in the Guarani population, and other Brazilian reference studies on infant-maternal health, such as the four Pelotas Birth Cohorts (1982, 1993, 2004 and 2015) and the Birth in Brazil Study. We also added questions based on the forms routinely used in health services for prenatal care, the hospital discharge summary after birth, the health card of the child, and the birth certificate. [file 12884_2020_3396_MOESM1_ESM.docx]

**COORTE DE NASCIMENTOS GUARANI - QUESTIONÁRIO PERINATAL**

ESTE QUESTIONÁRIO DEVE SER APLICADO A TODAS AS MULHERES RESIDENTES EM ALDEIAS PARTICIPANTES DO ESTUDO, EM FASE DE PUERPÉRIO OU PÓS-ABORTO. A ENTREVISTA DEVERÁ SER FEITA O MAIS PRECOCEMENTE POSSÍVEL, NOS PRIMEIROS 15 DIAS (DUAS SEMANAS) DO PÓS-PARTO OU ABORTO, OU O MAIS RÁPIDO POSSÍVEL. A ENTREVISTA DEVERÁ SERFEITA EM QUALQUER SITUAÇÃO, DESDE QUE NÃO HAJA RECUSA, E A QUALQUER TEMPO. EM CASO DE DÚVIDA, ENTRE EM CONTATO COM A COORDENAÇÃO DA PESQUISA. EM CASO DE GRAVIDEZ GEMELAR, ABRIR UM QUESTIONÁRIO PARA CADA CRIANÇA E INDICAR RN1 E RN2.

_____________________________________________________________________________________**BLOCO A - IDENTIFICAÇÃO**

**_____________________________________________________________________________________**

**P1 - DATA DA ENTREVISTA: ______/________/_________**

**NOME DO ENTREVISTADOR: _______________________________________________________**

**NOME COMPLETO DA MÃE: _______________________________________________________**

**SITUAÇÃO CONJUGAL**: ( ) COM MARIDO OU COMPANHEIRO ( ) SEM MARIDO OU COMPANHEIRO

**_____________________________________________________________________________________P2**

**DATA DE NASCIMENTO DA MÃE: ___/____/_______**Se anotada a idade, ir diretamente para a página **P4**

SENÃO SABE DATA DE NASCIMENTO, IR PARA A PÁGINA **P3**

**_____________________________________________________________________________________P3**

**IDADE DA MÃE: SE NÃO SABE A DATA DE NASCIMENTO, INFORMAR A IDADE: ______ ANOS**

**_____________________________________________________________________________________P4**

**POLO BASE DE RESIDÊNCIA: ________________________________________________**

**ALDEIA DE RESIDÊNCIA: _________________________________________**

**_____________________________________________________________________________________P5**

**A MÃE PODE SER ENTREVISTADA? ( )** SIM ( ) NÃO.

SE SIM **P9**

SE NÃO **P6**

**_____________________________________________________________________________________P6**

**POR QUÊ NÃO PODE SER ENTREVISTADA?** ( ) RECUSA ( ) ÓBITO ( ) HOSPITALIZADA COM RESTRIÇÃO DE ENTREVISTA ( ) OUTRO

SE RECUSA ENTREVISTA ENCERRADA

SE ÓBITO ou HOSPITALIZADA COM RESTRIÇÃO DE ENTREVISTA OU OUTRO IR PARA P7

**_____________________________________________________________________________________P7**

**A ENTREVISTA PODE SER REALIZADA COM INFORMANTE?**

( ) SIM ( ) NÃO, POR RECUSA ( ) NÃO, POR AUSÊNCIA( ) NÃO, OUTRO MOTIVO

SE NÃO, POR RECUSA ou NÃO, POR AUSÊNCIA ENTREVISTA ENCERRADA

**_____________________________________________________________________________________P8**

**QUAL A RELAÇÃO DO INFORMANTE COM A MÃE DA CRIANÇA?**

( ) PAIS ( ) COMPANHEIRO OU MARIDO ( ) FILHO ( ) OUTRO

**_____________________________________________________________________________________**

**BLOCO B - DEMOGRÁFICO E SOCIOECONÔMICO _____________________________________________________________________________________**

**P9**

**USA FOGO DE CHÃO?** ( ) SIM ( ) NÃO. SE NÃO P10

SE SIM, **QUAL A LOCALIZAÇÃO DESTE FOGO?** ( )DENTRO DE CASA ( )FORA DE CASA

SE DENTRO DE CASA**, ONDE FICA ESTE FOGO DENTRO DE CASA?**

( ) CÔMODO ÚNICO ( ) COZINHA ( ) QUARTO ( ) SALA ( )OUTRO

SE FORA DE CASA**, ONDE FICA ESTE FOGO FORA DE CASA?**

( )**NO TERRENO (A CÉU ABERTO)** ( )**COZINHA EXTERNA FECHADA** ( )**COZINHA EXTERNA ABERTA(SEM PAREDES)** ( )**VARANDA ABERTA** ( )**OUTRO**

**_____________________________________________________________________________________P10**

**USA FOGÃO À LENHA?** ( ) SIM ( ) NÃO. SE NÃO P11

SE SIM, **QUAL A LOCALIZAÇÃO DESTE FOGO?** ( )DENTRO DE CASA( )FORA DE CASA

SE DENTRO DE CASA**, ONDE FICA ESTE FOGO DENTRO DE CASA?**

( ) CÔMODO ÚNICO ( ) COZINHA ( ) QUARTO ( ) SALA ( )OUTRO

SE FORA DE CASA**, ONDE FICA ESTE FOGO FORA DE CASA?**

( )NO TERRENO (A CÉU ABERTO) ( )COZINHA EXTERNA FECHADA

( )COZINHA EXTERNA ABERTA(SEM PAREDES) ( )VARANDA ABERTA ( )OUTRO

**_____________________________________________________________________________________P11**

**USA FOGÃO A GÁS?** ( ) SIM ( ) NÃO. SE NÃO P12

SE SIM, **QUAL A LOCALIZAÇÃO DESTE FOGO?** ( )DENTRO DE CASA( )FORA DE CASA

SE DENTRO DE CASA**, ONDE FICA ESTE FOGO DENTRO DE CASA?**

( ) CÔMODO ÚNICO ( ) COZINHA ( ) QUARTO ( ) SALA ( )OUTRO

SE FORA DE CASA**, ONDE FICA ESTE FOGO FORA DE CASA?**

( )NO TERRENO (A CÉU ABERTO) ( )COZINHA EXTERNA FECHADA ( )COZINHA EXTERNA ABERTA(SEM PAREDES) ( )VARANDA ABERTA ( )OUTRO

**_____________________________________________________________________________________P12**

**QUAL FOGO VOCÊ MAIS USAPARA FAZER COMIDA?**

( )FOGO DE CHÃO ( ) FOGÃO À LENHA ( ) FOGÃO A GÁS ( ) OUTRO

**_____________________________________________________________________________________P13**

**ONDE OS MORADORES DA CASA COSTUMAM EVACUAR OU DEFECAR?**

( ) BANHEIRO OU SANITÁRIO DENTRO DE CASA

( ) BANHEIRO OU SANITÁRIO FORA DE CASA, USADO SOMENTE PELOS MORADORES DA CASA

( ) BANHEIRO OU SANITÁRIO FORA DE CASA, USADO TAMBÉM POR MORADORES DE OUTRAS CASAS DA ALDEIA

( ) BURACO PARA DEFECAR ( ) NO MATO ( ) OUTRO ( ) IGNORADO

se NO MATO ou OUTRO ou IGNORADO P14

**SE UMA DAS 3 OPÇÕES COM BANHEIRO OU SANITÁRIO:**

**ONDE O ESGOTO DO BANHEIRO OU SANITÁRIO É LANÇADO?**

( ) REDE GERAL PÚBLICA DE ESGOTO ( ) FOSSA CONSTRUÍDA POR ÓRGÃO PÚBLICO (FUNASA, SESAI, OUTRO) ( ) FOSSA CONSTRUÍDA PELOS MORADORES DO DOMICÍLIO ( ) VALA ABERTA

( ) DIRETO PARA O RIO, LAGO, AÇUDE OU MAR ( ) OUTRO ( ) IGNORADO

**_____________________________________________________________________________________P14**

**QUAL O PRINCIPAL PONTO ONDE OS MORADORES DA SUA CASA PEGAM ÁGUA PARA USO DOMÉSTICO (POR EXEMPLO, LAVAR LOUÇA, COZINHAR, TOMAR BANHO, ETC.)?**

( ) TORNEIRA OU BICA (TAQUARA, MANGUEIRA, CANO) DENTRO DE CASA

( )TORNEIRA OU BICA (TAQUARA, MANGUEIRA, CANO) FORA DE CASA USADA SOMENTE PELOS MORADORES DA CASA

( ) TORNEIRA OU BICA (TAQUARA, MANGUEIRA, CANO) FORA DE CASA USADA TAMBÉM POR MORADORES DE OUTRAS CASAS (COLETIVA)

( ) DIRETO DA NASCENTE, OLHO D'AGUA OU POÇO NA ALDEIA

( ) DIRETO DA CACHOEIRA,RIO,AÇUDE, REPRESA OU LAGO

( ) OUTRO ( ) IGNORADO

se DIRETO NA CACHOEIRA, RIO, AÇUDE, REPRESA, LAGO ou DIRETO DA NASCENTE, OLHO D'AGUA OU POÇO ou OUTRO **P15**

**SE UMA DAS 3 OPÇÕES COM TORNEIRA OU BICA:**

**EM CASO DE TORNEIRA OU BICA (TAQUARA, MANGUEIRA, CANO), QUAL A FORMA DE ABASTECIMENTO?**

( ) REDE GERAL DE DISRTIBUIÇÃO PÚBLICA

( ) REDE LOCAL DE DISTRIBUIÇÃO NA ALDEIA (SISTEMA LOCAL CONSTRUÍDO POR ÓRGÃO PÚBLICO, COMO FUNASA, SESAI OU OUTRO)

( ) DIRETO DA NASCENTE, OLHO D'AGUA OU POÇO NA ALDEIA

( ) DIRETO DA CACHOEIRA,RIO, AÇUDE, REPRESA OU LAGO

( ) OUTRO ( ) IGNORADO

**_____________________________________________________________________________________P15**

**ONDE PEGA ÁGUA PARA BEBER?**

( ) TORNEIRA OU BICA (TAQUARA, MANGUEIRA, CANO) DENTRO DE CASA

( ) TORNEIRA OU BICA (TAQUARA, MANGUEIRA, CANO) FORA DE CASA USADA SOMENTE PELOS MORADORES DA CASA

( ) TORNEIRA OU BICA (TAQUARA, MANGUEIRA, CANO) FORA DE CASA USADA TAMBÉM POR MORADORES DE OUTRAS CASAS (COLETIVA)

( ) DIRETO DA NASCENTE, OLHO D'AGUA OU POÇO NA ALDEIA

( ) DIRETO DA CACHOEIRA,RIO, AÇUDE, REPRESA OU LAGO

( ) OUTRO ( ) IGNORADO

**NA SUA CASA É COSTUME GUARDAR ÁGUA PARA BEBER EM PANELA, CACIMBA, FILTRO, MORINGA OU OUTRO RECIPIENTE?** ( ) SIM ( ) NÃO

**A ÁGUA GUARDADA PARA BEBER É TRATADA PELOS MORADORES DA CASA (COLOCAÇÃO DE CLORO, ÁGUA SANITÁRIA OU HIPOCLORITO DE SÓDIO, FILTRADA, FERVIDA OU COADA)?**

( ) SIM ( ) NÃO ( ) IGNORADO

**_____________________________________________________________________________________P16**

**O QUE É FEITO COM O LIXO DA SUA CASA?**

( ) COLETADO DIRETAMENTE POR SERVIÇO PÚBLICO DE LIMPEZA NA PRÓPRIA CASA

( ) COLOCADO EM CAÇAMBA OU LIXEIRA COLETIVA, ESVAZIADA PERIODICAMENTE POR SERVIÇO PÚBLICO DE LIMPEZA

( ) QUEIMADO OU ENTERRADO NA ALDEIA/JOGADO NO TERRENO DA ALDEIA (A CÉU ABERTO)

( ) OUTRO ( ) IGNORADO

**_____________________________________________________________________________________P17**

**EXISTE ENERGIA ELÉTRICA (LUZ) NA SUA CASA?** ( ) SIM ( ) NÃO

SE SIM, **QUAL A FONTE DA ENERGIA ELÉTRICA?**

( ) REDE ELÉTRICA DE COMPANHIA DISTRIBUIDORA ( ) GERADOR ( ) PLACA SOLAR ( )OUTRO( ) IGNORADO

**_____________________________________________________________________________________P18**

**POSSUI:**

**RÁDIO?**( ) SIM ( ) NÃO

**se sim, QUAL A QUANTIDADE? _____**

**GELADEIRA?** ( ) SIM ( ) NÃO

**se sim, QUAL A QUANTIDADE? _____**

**FREEZER?** ( ) SIM ( ) NÃO

**se sim, QUAL A QUANTIDADE? _____**

**DVD/BLUE RAY?** ( ) SIM ( ) NÃO

**se sim, QUAL A QUANTIDADE? _____**

**FOGÃO A GAS?** ( ) SIM ( ) NÃO

**se sim, QUAL A QUANTIDADE? _____**

**_____________________________________________________________________________________P19**

**FILTRO/PURIFICADOR DE ÁGUA?** ( ) SIM ( ) NÃO

**se sim, QUAL A QUANTIDADE? _____**

**MÁQUINA DE LAVAR ROUPA?** ( ) SIM ( ) NÃO

**se sim, QUAL A QUANTIDADE? _____**

**FORNO DE MICROONDAS/FORNO ELÉTRICO?** ( ) SIM ( ) NÃO

**se sim, QUAL A QUANTIDADE? _____**

**LINHA DE TELEFONE FIXO?** ( ) SIM ( ) NÃO

**se sim, QUAL A QUANTIDADE? _____**

**_____________________________________________________________________________________P20**

**TELEFONE CELULAR** ( ) SIM ( ) NÃO

**se sim, QUAL A QUANTIDADE? _____**

**COMPUTADOR/TABLET?** ( ) SIM ( ) NÃO

**se sim, QUAL A QUANTIDADE? _____**

**MOTOCICLETA?** ( ) SIM ( ) NÃO

**se sim, QUAL A QUANTIDADE? _____**

**ANIMAL DE CARGA/TRABALHO?** ( ) SIM ( ) NÃO

**se sim, QUAL A QUANTIDADE? _____**

**_____________________________________________________________________________________P21**

**POSSUI TELEVISÃO?** ( ) SIM ( ) NÃO

**se sim, QUAL A QUANTIDADE? _____**

**AUTOMÓVEL?** ( ) SIM ( ) NÃO

**se sim, QUAL A QUANTIDADE? _____**

**ANTENA PARABÓLICA?** ( ) SIM ( ) NÃO

**se sim, QUAL A QUANTIDADE? _____**

**BICICLETA?** ( ) SIM ( ) NÃO

**se sim, QUAL A QUANTIDADE? _____**

**_____________________________________________________________________________________**

**P22**

**INDIQUE AS FONTES DE ALIMENTOS CONSUMIDOS EM SUA CASA NO ÚLTIMO MÊS:** (RESPOSTA MÚLTIPLA)

( ) PLANTAÇÃO OU CRIAÇÃO DE ANIMAIS

( ) CAÇA E PESCA

( ) COLETA

( ) TROCA OU DOAÇÃO DENTRO DA ALDEIA

( ) DOAÇÕES DE FORA DA ALDEIA

( ) COMPRA

**_____________________________________________________________________________________P23**

**QUAL É A PRINCIPAL FONTE DE COMIDA DA CASA?** ( ) PLANTAÇÃOOU CRIAÇÃO DE ANIMAIS ( ) CAÇA E PESCA ( ) COLETA ( ) TROCA OU DOAÇÃO DENTRO DA ALDEIA ( ) DOAÇÕES DE FORA DA ALDEIA ( ) COMPRA ( ) OUTROS

**A FAMÍLIA RECEBEU CESTAS BÁSCIAS NO ÚLTIMO MÊS?** ( ) SIM ( ) NÃO ( ) IGNORADO

se NÃO ou IGNORADO **P24**

se sim, **QUANTAS? ____**

**_____________________________________________________________________________________P24**

**qual foi o último ano DE ESTUDO completo cursado PELA MÃE com aprovação?**

**( )** Nunca estudou

( ) Estudou, mas não sabe informar o grau de instrução

( ) Estudou, mas nenhum ano foi concluído

( ) Alfabetização/1º ano/EJA-Alfabetização

( ) 1ª série/2º ano/EJA-Série iniciais

( ) 2ª série/3º ano/EJA-Série iniciais

( ) 3ª série/4º ano/EJA-Série iniciais

( ) 4ª série/5º ano/EJA-Série iniciais

( ) 5ª série/6º ano/EJA-Série finais

( ) 6ª série/7º ano/EJA-Série finais

( ) 7ª série/8º ano/EJA-Série finais

( ) 8ª série/9º ano/EJA-Série finais

( ) 1º ano (2º grau ou Ensino Médio)/EJA-Médio

( ) 2º ano (2º grau ou Ensino Médio)/EJA-Médio

( ) 3º ano (2º grau ou Ensino Médio)/EJA-Médio

( ) Graduação

( ) Pós-Graduação

**____________________________________________________________________________________BLOCO C: ENTREVISTA SOBRE GESTAÇÃO, PARTO, PUERPÉRIO E RECÉM-NASCIDO**

**_____________________________________________________________________________________P25**

**ONDE FOI REALIZADO O PARTO DO RECÉM NASCIDO?**

( ) ALDEIA ( ) HOSPITAL ( ) CASAI ( ) OUTRO ( ) IGNORADO

**QUEM FEZ O PARTO?** (  **)** MÉDICO ( ) ENFERMEIRO ( ) PARTEIRA ( ) AGENTE INDÍGENA DE SAÚDE ( ) PARTO NÃO ASSISTIDO (SOZINHA) ( ) OUTRO ( ) IGNORADO

**_____________________________________________________________________________________P26**

**HOUVE ACOMPANHAMENTO POR PARTEIRA DURANTE A GRAVIDEZ? ( )** SIM ( ) NÃO ( )IGNORADO

**_____________________________________________________________________________________P27**

**A MÃE FUMOU CIGARRO DE JURUÁ DURANTE A GRAVIDEZ?**

**( )** SIM ( ) NÃO ( ) IGNORADO

se NÃO ou IGNORADO **P29**

se SIM, **FUMOU DURANTE TODA A GRAVIDEZ? ( )** SIM ( ) NÃO ( )IGNORADO

se SIM **P28**

se NÃO ou IGNORADO,

**FUMOU NOS 3 PRIMEIROS MESES DE GRAVIDEZ? (PRIMEIRO TRIMESTRE)**

**( )** SIM ( ) NÃO ( )IGNORADO

**FUMOU DOS 4 AOS 6 MESES DE GRAVIDEZ? (SEGUNDO TRIMESTRE)**

**( )** SIM ( ) NÃO ( )IGNORADO

**FUMOU DOS 7 MESES AO FIM DA GRAVIDEZ? (TERCEIRO TRIMESTRE)**

**( )** SIM ( ) NÃO ( )IGNORADO

**_____________________________________________________________________________________P28**

**DURANTE TODO O PERÍODO QUE FUMOU NA GRAVIDEZ, FUMAVA:**

( ) DIARIAMENTE ( ) SEMANALMENTE, MAS NEM TODO DIA ( ) MENSALMENTE, MAS NEM TODA SEMANA ( ) EVENTUALMENTE ( ) IGNORADO

**QUANTOS CIGARROS FUMAVA POR DIA? (UM MAÇO=20 CIGARROS): _____**

**_____________________________________________________________________________________P29**

**A MÃE BEBEU BEBIDA ALCOÓLICA DURANTE A GRAVIDEZ?**

**( )** SIM ( ) NÃO ( )IGNORADO

se NÃO ou IGNORADO **P32**

se SIM, **BEBEU DURANTE TODA A GRAVIDEZ?**

**( )** SIM ( ) NÃO ( )IGNORADO

se SIM **P31**

se NÃO ou IGNORADO **P30**

**_____________________________________________________________________________________P30**

**BEBEU BEBIDA ALCOÓLICA:**

**NOS 3 PRIMEIROS MESES DE GRAVIDEZ? (PRIMEIRO TRIMESTRE)**

**( )** SIM ( ) NÃO ( )IGNORADO

**DOS 4 AOS 6 MESES DE GRAVIDEZ? (SEGUNDO TRIMESTRE)**

**( )** SIM ( ) NÃO ( )IGNORADO

**DOS 7 MESES AO FIM DA GRAVIDEZ? (TERCEIRO TRIMESTRE)**

**( )** SIM ( ) NÃO ( )IGNORADO

**_____________________________________________________________________________________P31**

**QUAL O PRINCIPAL TIPO DE BEBIDA CONSUMIDO DURANTE A GRAVIDEZ?**

( ) VINHO ( ) CERVEJA ( ) DESTILADA (CAHAÇA, VODKA, WHISKY, RUM ETC.)

**NO PERÍODO QUE BEBEU BEBIDA ALCOÓLICA DURANTE A GRAVIDEZ, BEBIA:**

( ) 1 VEZ AO MÊS OU MENOS ( ) 2 A 4 VEZES POR MÊS ( ) 2 A 3 VEZES POR SEMANA

( ) 4 VEZES O MAIS POR SEMANA ( ) IGNORADO

**_____________________________________________________________________________________SOBRE O RECEM-NASCIDO**

**_____________________________________________________________________________________**

**P32**

**COM QUANTO TEMPO DE VIDA A CRIANÇA MAMOU PELA PRIMEIRA VEZ NO PEITO DA MÃE?**

( ) NA 1^a^ HORA DE VIDA ( ) DEPOIS DA 1^a^ HORA DE VIDA ATÉ < 24HORAS

( ) 2 ^O^ dia ( ) 3 ^O^ dia ( ) 4 ^O^ dia ( ) 5 ^O^ dia ( ) 6 ^O^ dia ( ) 7 ^O^ dia ( ) 8 ^O^DIA OU MAIS

( ) Ainda não mamou

**A CRIANÇA JÁ MAMOU NO PEITO DE OUTRA MULHER?**

**( )** SIM ( ) NÃO ( )IGNORADO

se SIM, **COM QUANTO TEMPO DE VIDA A CRIANÇA COMEÇOU A MAMAR NO PEITO DE OUTRA MULHER?**

( ) NA 1^a^ HORA DE VIDA ( ) DEPOIS DA 1^a^ HORA DE VIDA ATÉ < 24HORAS

( ) 2 ^O^ dia ( ) 3 ^O^ dia ( ) 4 ^O^ dia ( ) 5 ^O^ dia ( ) 6 ^O^ dia ( ) 7 ^O^ dia ( ) 8 ^o^dia ou mais

**ENQUANTO OUTRA MULHER DEU DE MAMAR, A MÃE CONTINUOU DANDO O PEITO PARA A CRIANÇA?**

**( )** SIM ( ) NÃO ( )IGNORADO

**_____________________________________________________________________________________P33**

**A CRIANÇA JÁ BEBEU ÁGUA, CHÁ, SUCO DE FRUTA OU BEBIDA TRADICIONAL?**

**( )** SIM ( ) NÃO ( )IGNORADO

**A CRIANÇA JÁ BEBEU LEITE EM PÓ (NAN, NINHO, NESTOGENO, TODDY, NESCAU, OUTRO), LEITE DE VACA, DE CABRA, OU OUTRO LEITE NÃO HUMANO?**

**( )** SIM ( ) NÃO ( )IGNORADO

**_____________________________________________________________________________________P34**

**DESDE QUE NASCEU, A CRIANÇA USOU MAMADEIRA? ( )** SIM ( ) NÃO ( )IGNORADO

**DESDE QUE NASCEU, A CRIANÇA USOU BICO/CHUPETA? ( )** SIM ( ) NÃO ( )IGNORADO

**O PAI DA CRIANÇA TEM ASMA? ( )** SIM ( ) NÃO ( )IGNORADO

**A MÃE DA CRIANÇA TEM ASMA? ( )** SIM ( ) NÃO ( )IGNORADO

**_________________________________________________________________________________**

**P35**

**OBSERVAR O TIPO DE CHÃO PREDOMINANTE.**

**( )** TERRA ( ) MADEIRA ( ) CIMENTO ( ) PISO ( ) OUTRO ( ) IGNORADO

**OBSERVAR SE USA ALGUM OUTRO MATERIAL PARA FORRAR O CHÃO.**

( ) NÃO USA ( ) TERRA ( ) MADEIRA ( ) CIMENTO ( ) PISO

( ) LONA OU PLÁSTICO ( ) OUTRO ( ) IGNORADO

**OBSERVAR O TIPO DE PAREDE PREDOMINANTE.**

( )PALHA ( ) TRONCOS ( ) TÁBUA ( ) PAU-À-PIQUE OU ADOBE (BARRO E MADEIRA) ( ) ALVENARIA ( ) LONA OU PLÁSTICO ( ) LENÇOL, COBERTOR, PANO ( ) OUTRO ( ) IGNORADO

**OBSERVAR SE USA ALGUM OUTRO MATERIAL PARA FORRAR A PAREDE.**

( )PALHA ( ) TRONCOS ( ) TÁBUA ( ) PAU-À-PIQUE OU ADOBE (BARRO E MADEIRA) ( ) ALVENARIA ( ) LONA OU PLÁSTICO ( ) LENÇOL, COBERTOR, PANO ( ) OUTRO ( ) IGNORADO

**OBSERVAR O TIPO DE TETO PREDOMINANTE:**

( )PALHA( ) TAQUARA( ) RIPA DE MADEIRA ( ) TÁBUA( ) LAJE ( ) TELHA DE BARRO ( ) TELHA DE ZINCO OU AMIANTO (TIPO "ETERNIT") ( ) LONA OU PLÁSTICO

( ) OUTRO ( ) IGNORADO

**_____________________________________________________________________________________**

**P36**

**OBSERVAR SE USA ALGUM OUTRO MATERIAL PARA FORRAR O TETO.**

( ) PALHA( ) TAQUARA ( ) RIPA DE MADEIRA ( ) TÁBUA ( ) LAJE ( ) TELHA DE BARRO ( ) TELHA DE ZINCO OU AMIANTO (TIPO "ETERNIT") ( ) LONA OU PLÁSTICO

( ) OUTRO ( ) IGNORADO

**COMO É A DIVISÃO DE CÔMODOS DA CASA?**

( )CÔMODO ÚNICO SEM VARANDA ( ) CÔMODO ÚNICO COM VARANDA

( ) MAIS DE UM CÔMODO SEM VARANDA ( ) MAIS DE UM CÔMODO COM VARANDA

SE CÔMODO ÚNICO **P38**

SE MAIS DE UM CÔMODO **P37**

**____________________________________________________________________________________P37**

**QUANTOS QUARTOS? _________**

**QUANTAS SALAS? _________**

**QUANTOS BANHEIROS? _________**

**QUANTAS COZINHAS? _________**

**QUANTAS PORTAS EXTERNAS TEM A CASA? _________**

**QUANTAS JANELAS TEM A CASA? _________**

**_____________________________________________________________________________________DADOS SECUNDARIOS: Os dados secundários devem ser retirados do cartão da gestante, do prontuário materno, cópia da DO de morte fetal, cópia da DNV, do cartão da criança ou de outra fonte disponível.**

**_____________________________________________________________________________________P38**

**TIPO DE GRAVIDEZ:** ( ) ÚNICA ( ) GEMELAR

se GEMELAR, **QUANTOS GÊMEOS? _______**

**DESFECHO DESSA GRAVIDEZ:** ( ) NASCIDO VIVO ( ) NATIMORTO ( ) ABORTO

**_____________________________________________________________________________________P39**

**A MÃE FEZ ALGUMA CONSULTA DE PRÉ-NATAL NESTA GRAVIDEZ?**

**( )** SIM ( ) NÃO ( )IGNORADO

se NÃO ou IGNORADO P56

**INDIQUE AS FONTES UTILIZADAS PARA RESPONDER AS PERGUNTAS SOBRE O PRÉ-NATAL E A CRIANÇA (RESPOSTA MÚLTIPLA):**

( ) CARTÃO DA GESTANTE

( ) PRONTUÁRIO MATERNO/CRIANÇA

( ) CADERNETA DE VACINA/ESPELHO

( ) CADERNETA DE SAÚDE DA CRIANÇA

( ) CADERNETA DE SAÚDE DO ADULTO

( ) REGISTROS DA EQUIPE MULTIDISCIPLINAR

( ) CÓPIA DA DECLARAÇÃO DE ÓBITO (DO) (MORTE FETAL)

( ) CÓPIA DA DECLARAÇÃO DE NASCIDO VIVO (DN)

( ) OUTRA FONTE

**ONDE FEZ AS CONSULTAS DE PRÉ-NATAL (MARCAR O LOCAL PRINCIPAL DE REALIZAÇÃO).**

**( )** ALDEIA **( )** FORA DA ALDEIA

**( )** DENTRO E FORA DA ALDEIA SEM PREDOMINÂNCIA DE UM OU OUTRO.

se NA ALDEIA **P40**

**FORA DA ALDEIA, ONDE? ____________________________________________________**

**_____________________________________________________________________________________P40**

**DATA DA ÚLTIMA MENSTRUAÇÃO: ____/_____/_________**.

**ULTRASSONOGRAFIA OBSTÉTRICA**

**( )** SOLICITADO E REALIZADO **( ) S**OLICITADO E NÃO REALIZADO **( ) S**OLICITADO E IGNORADO QUANTO À REALZIAÇÃO **( )** NÃO SOLICITADO **( )** IGNORADO

**DATA DA PRIMEIRA USG DURANTE A GESTAÇÃO** **____/_____/_________**.

**IDADE GESTACIONAL EM SEMANAS, NA PRIMEIRA CONSULTA PRÉ-NATAL (DUM OU USG OU ALTURA UTERINA):_______**

**QUANTAS CONSULTAS DE PRÉ-NATAL FEZ DURANTE A GRAVIDEZ** (COM MÉDICO OU ENFERMEIRO): ______**_______________________________________________________________________________HISTÓRICO OBSTÉTRICO**

**_____________________________________________________________________________________P41**

**NÚMERO DE GESTAÇÕES (INCLUINDO A ÚLTIMA GESTAÇÃO): _____**

**NÚMERO DE ABORTOS (ANTES DA ÚLTIMA GESTAÇÃO):**____

**NÚMERO DE PARTOS (ANTES DA ÚLTIMA GESTAÇÃO): _____**

**QUANTOS NASCERAM VIVOS (ANTES DA ÚLTIMA GESTAÇÃO)?** _____

**QUANTOS NASCERAM MORTOS (ANTES DA ÚLTIMA GESTAÇÃO)?** _____

**_____________________________________________________________________________________P42**

**PESO DA MÃE ANTES DA GRAVIDEZ (em kg)**. ____**,___ (3 CARACTERES)**

**DATA DO PESO ANTES DA GRAVIDEZ ____/_____/_________**

**ULTIMO PESO DA MÃE, ANTES DO PARTO**. ____**,___ (3 CARACTERES)**

**DATA DO ÚLTIMO PESOANTES DO PARTO ____/_____/_________.**

**_____________________________________________________________________________________P43**

**ESTATURA DA MÃE (A MAIS RECENTE) (EM CM)** _______ **,___** .

**DATA DA ESTATURA ____/_____/_________.**

**_____________________________________________________________________________________P44**

**HOUVE PRESCRIÇÃO DE SULFATO FERROSO (PELO MENOS 1 COMPRIMIDO POR DIA) A PARTIR DA PRIMEIRA CONSULTA DE PRÉ-NATAL ATÉ O FINAL DA GRAVIDEZ?**

**( )** SIM ( ) NÃO ( )IGNORADO

MÊS DE GESTAÇÃO DA PRIMEIRA PRESCRIÇÃO DE SULFATO FERROSO: ____.

NÚMERO DE MESES DE GESTAÇÃO COBERTOS COM PRESCRIÇÃO DE SULFATO FERROSO: ____.

**_____________________________________________________________________________________**

**P45**

**EXAMES SOLICITADOS NO PRÉ-NATAL**

**TIPO SANGUINEO (ABO) + Rh:** ( ) SOLICITADO E REALIZADO ( ) SOLICITADO E NÃO REALIZADO ( ) SOLICITADO E IGNORADO QUANTO À REALIZAÇÃO ( ) NÃO SOLICITADO ( ) IGNORADO

**HEMOGRAMA:** ( ) SOLICITADO E REALIZADO ( ) SOLICITADO E NÃO REALIZADO ( ) SOLICITADO E IGNORADO QUANTO À REALIZAÇÃO ( ) NÃO SOLICITADO ( ) IGNORADO

**GLICEMIA DE JEJUM:** ( ) SOLICITADO E REALIZADO ( ) SOLICITADO E NÃO REALIZADO ( ) SOLICITADO E IGNORADO QUANTO À REALIZAÇÃO ( ) NÃO SOLICITADO ( ) IGNORADO

**TESTE RÁPIDO PARA SÍFILIS E/OU VDRL:** ( ) SOLICITADO E REALIZADO ( ) SOLICITADO E NÃO REALIZADO ( ) SOLICITADO E IGNORADO QUANTO À REALIZAÇÃO ( ) NÃO SOLICITADO ( ) IGNORADO

**URINA 1:** ( ) SOLICITADO E REALIZADO ( ) SOLICITADO E NÃO REALIZADO ( ) SOLICITADO E IGNORADO QUANTO À REALIZAÇÃO ( ) NÃO SOLICITADO ( ) IGNORADO

**URINOCULTURA:** ( ) SOLICITADO E REALIZADO ( ) SOLICITADO E NÃO REALIZADO ( ) SOLICITADO E IGNORADO QUANTO À REALIZAÇÃO ( ) NÃO SOLICITADO ( ) IGNORADO

**TESTE RÁPIDO ANTI-HIV OU ANTI-HIV:** ( ) SOLICITADO E REALIZADO ( ) SOLICITADO E NÃO REALIZADO ( ) SOLICITADO E IGNORADO QUANTO À REALIZAÇÃO ( ) NÃO SOLICITADO ( ) IGNORADO

**_____________________________________________________________________________________P46**

**HBsAg:** ( ) SOLICITADO E REALIZADO ( ) SOLICITADO E NÃO REALIZADO ( ) SOLICITADO E IGNORADO QUANTO À REALZIAÇÃO ( ) NÃO SOLICITADO ( ) IGNORADO

**TOXOPLASMOSE:** ( ) SOLICITADO E REALIZADO ( ) SOLICITADO E NÃO REALIZADO ( ) SOLICITADO E IGNORADO QUANTO À REALZIAÇÃO ( ) NÃO SOLICITADO ( ) IGNORADO

**EXAME PARASITOLÓGICO DE FEZES:** ( ) SOLICITADO E REALIZADO ( ) SOLICITADO E NÃO REALIZADO ( ) SOLICITADO E IGNORADO QUANTO À REALZIAÇÃO ( ) NÃO SOLICITADO ( ) IGNORADO

**_____________________________________________________________________________________P47**

**TOMOU, PELO MENOS, 3 DOSES DE VACINA CONTRA TÉTANO ANTES DO ÚLTIMO PARTO?**

**( )** SIM ( ) NÃO ( )IGNORADO)

**TOMOU, PELO MENOS, UMA DOSE DE dT NOS ÚLTIMOS CINCO ANOS?**

**( )** SIM ( ) NÃO ( )IGNORADO

**TOMOU, PELO MENOS, 3 DOSES DE VACINA CONTRA HEPATITE B ANTES DO ÚLTIMO PARTO?**

**( )** SIM ( ) NÃO ( )IGNORADO

**REALIZOU EXAME ODONTOLÓGICO DURANTE A GRAVIDEZ?**

**( )** SIM ( ) NÃO ( )IGNORADO

**____________________________________________________________________________________**

**P48**

**DURANTE A GRAVIDEZ, EXISTE REGISTRO DE:**

**PRESSÃO ARTERIAL**? **( )** SIM ( ) NÃO.

Se sim. Quantos? **______**

**ALTURA**? **( )** SIM ( ) NÃO.

**PESO? ( )** SIM ( ) NÃO.

Se sim. Quantos? **______**

**_____________________________________________________________________________________P49**

**ALTURA UTERINA? ( )** SIM ( ) NÃO.

Se sim. Quantos? **______**

**BATIMENTO CARDÍACO FETAL**? **( )** SIM ( ) NÃO.

Se sim. Quantos? **______**

**EXAME DAS MAMAS**? **( )** SIM ( ) NÃO.

Se sim. Quantos? **______**

**_____________________________________________________________________________________P50**

**FOI REALIZADO TESTE RÁPIDO ANTI-HIV NA MÃE, DURANTE O TRABALHO DE PARTO?**

**( )** SIM ( ) NÃO ( )IGNORADO

**FOI REALIZADO O TESTE VDRL NA MÃE, DURANTE O TRABALHO DE PARTO?**

**( )** SIM ( ) NÃO ( )IGNORADO

**TIPO DE PARTO ( )** VAGINAL ( ) CESARIANA

Se vaginal **P52**

**_____________________________________________________________________________________P51**

**QUAL FOI A INDICAÇÃO DA CESARIANA?**

( ) SOFRIMENTO FETAL

( ) DESPROPORÇÃO CÉFALO-PÉLVICA

( ) DISTÓCIA DE APRESENTAÇÃO

( ) HEMORRAGIA MATERNA

( ) PARADA DE PROGRESSÃO

( ) ECLÂMPSIA

( ) PRÉ-ECLÂMPSIA

( ) PÓS-MATURIDADE

( ) MORTE FETAL

( ) DIABETES MATERNA

( ) REPETIÇÃO "CASO O PARTO ANTERIOR TENHA SIDO CESÁREA''

( ) LAQUEADURA TUBÁRIA

( ) OUTRO

**_____________________________________________________________________________________P52**

**INDIQUE SE OCORREU DURANTE O TRABALHO DE PARTO: (MÚLTIPLA ESCOLHA)**

( ) INDUÇÃO (OCITOCINA)

( ) ANALGESIA

( ) EPISIOTOMIA

( ) FÓRCEPS

( ) NÃO APRESENTOU NENHUM DOS EVENTOS ACIMA LISTADOS

**_____________________________________________________________________________________P53**

**INDIQUE QUAIS DESSES EVENTOS FAZEM PARTE DA HISTÓRIA PATOLÓGICA PREGRESSA E INTERCORRÊNCIAS DURANTE GESTAÇÃO E PARTO (MÚLTIPLA ESCOLHA)**.

( ) HIPERTENSÃO PRÉ-GESTACIONAL

( ) HIPERTENSÃO NA GESTAÇÃO

( ) PRÉ-ECLAMPSIA

( ) ECLAMPSIA

( ) CARDIOPATIA

( ) DIABETES PRÉ-GESTACIONAL

( ) DIABETES NA GESTAÇÃO

( ) NÃO APRESENTOU NENHUM DOS EVENTOS ACIMA LISTADOS

**_____________________________________________________________________________________P54**

( ) INFECÇAO URINÁRIA NA GESTAÇÃO

( ) AMEAÇA DE PARTO PREMATURO

( ) DESPROPORÇÃO CÉFALO PÉLVICA

( ) HEMORRAGIA NO PRIMEIRO TRIMESTRE

( ) HEMORRAGIA NO SEGUNDO TRIMESTRE

( ) HEMORRAGIA NO TERCEIRO TRIMESTRE

( ) NÃO APRESENTOU NENHUM DOS EVENTOS ACIMA LISTADOS

**_____________________________________________________________________________________P55**

( ) ANEMIA CRÔNICA

( ) RUPTURA PREMATURA DE MEMBRANA

( ) CIRCULAR DE CORDÃO

( ) ASFIXIA NEONATAL

( ) ASPIRAÇÃO DE MECÔNIO

( ) NÃO APRESENTOU NENHUM DOS EVENTOS ACIMA LISTADOS

**_____________________________________________________________________________________P56**

**NOME DA CRIANÇA ______________________________________________________**

**DATA DE NASCIMENTO: ___/_____/_________.**

**SEXO DA CRIANÇA: ( )** MASCULINO ( ) FEMININO

**PESO AO NASCER (EM GRAMAS): _________g (**4 CARACTERES**)**

**COMPRIMENTO (EM CM): _____,__cm (**3 CARACTERES**)**

**PERÍMETRO CEFÁLICO (EM CM): ______, ___ cm (**3 CARACTERES**)**

**_____________________________________________________________________________________P57**

**HÁ IG (CAPURRO)?** ( ) SIM ( ) NÃO ( ) IGNORADO

INFORMAR IDADE GESTACIONAL NO NASCIMENTO: _______SEMANAS

**RELAÇÃO PESO/IDADE GESTACIONAL** ( ) ADEQUADO(AIG) ( ) PEQUENO(PIG) ( ) GRANDE(GIG) ( ) IGNORADO

**APGAR** 1’: ______. ( ) IGNORADO

**APGAR 5’: _______.** ( ) IGNORADO

**REANIMAÇÃO?** ( ) SIM ( ) NÃO ( ) IGNORADO

**________________________________________________________________________________**

**P58**

**PATOLOGIAS OBSERVADAS (MÚLTIPLA ESCOLHA):**

( ) MEMBRANA HIALINA

( ) BRONCODISPLASIA PULMONAR

( ) ASFIXIA NEONATAL

( ) NEUROLÓGICAS

( ) HEMORRAGIA

( ) NÃO APRESENTOU NENHUM DOS EVENTOS ACIMA LISTADOS

**_____________________________________________________________________________________P59 (MÚLTIPLA ESCOLHA)**

( ) ICTERÍCIA NEONATAL/HIPERBILIRRUBINEMIA

( ) INFECÇÃO CONGENITA

( ) SEPSE NEONATAL

( ) ANOMALIA CONGÊNITA

( ) NÃO APRESENTOU NENHUM DOS EVENTOS ACIMA LISTADOS

**O RN PRECISOU DE UTI NEONATAL?** ( ) SIM ( ) NÃO ( ) IGNORADO

**_____________________________________________________________________________________P60**

**FOI REALIZADO O TESTE DO PEZINHO NO RECÉM NASCIDO?**

( ) SIM ( ) NÃO ( ) IGNORADO

**FOI REALIZADO O TESTE DA ORELHINHA NO RECÉM NASCIDO?**

( ) SIM ( ) NÃO ( ) IGNORADO

**FOI REALIZADO O TESTE DO OLHINHO NO RECEM NASCIDO?**

( ) SIM ( ) NÃO ( ) IGNORADO

**O RN FALECEU ANTES DESSA ENTREVISTA?** ( ) SIM ( ) NÃO

**SE SIM, encerrar entrevista e gerar alerta para preencher questionário FINAL e concluir a pesquisa com a criança.**

**_____________________________________________________________________________________P61**

**QUANTAS PESSOAS MORAM NA SUA CASA (INCLUINDO O RECÉM NASCIDO? _______.**

**QUANTAS CRIANÇAS MENORES DE CINCO ANOS MORAM NA SUA CASA (INCLUINDO O RECÉM NASCIDO)?** _______.

**QUANTAS PESSOAS COM 10 ANOS OU MAIS MORAM NA SUA CASA (MARCAR NO BOTÃO ESCALA)? _____.**

**_____________________________________________________________________________________PARA CADA MORADOR COM 10 ANOS OU MAIS DE IDADE _____________________________________________________________________________________P62**

**PRIMEIRO NOME: _____________________________________________**

**SEXO** ( ) MASCULINO ( ) FEMININO

**IDADE: _______** anos**.**

**GRAU DE PARENTESCO COM O RECÉM NASCIDO:**

( )PAIS ( ) AVÓS ( ) IRMÃOS ( )OUTROS

**OCUPAÇÃO:** ( )SEM OCUPAÇÃO ( ) PROFESSOR ( ) AGENTE INDÍGENA DE SAÚDE (AIS OU AISAN) ( ) MERENDEIRA ( ) VIGILANTE ( ) PEDREIRO ( ) ENFERMEIRO ( ) AGRICULTOR ( ) TÉCNICO DE ENFERMAGEM OU DE HIGIENE DENTAL ( ) MOTORISTA ( ) TÉCNICO FUNAI ( ) APOSENTADO ( ) OUTROS

**LOCAL DE TRABALHO:** ( )DENTRO DA ALDEIA ( ) FORA DA ALDEIA ( ) NÃO SE APLICA

**RENDA REGULAR NO ÚLTIMO MÊS (SALÁRIO DE EMPREGO FORMAL):**

( ) NÃO SE APLICA ( ) ATÉ R$200

( ) R$201-R$400 ( ) R$401-R$600

( ) R$601-R$800 ( ) R$801-R$1000

( ) R$1001-R$1200 ( ) R$1201-R$1400

( ) R$1401-R$1600 ( ) R$1601-R$1800

( ) R$1801-R$2000 ( ) R$2001-R$2500

( ) R$2501-R$3000 ( ) R$3001 OU MAIS

**APOSENTADORIAS E PENSÕES (INSS, FUNRURAL, OUTROS):**

( ) NÃO SE APLICA ( ) ATÉ R$200

( ) R$201-R$400 ( ) R$401-R$600

( ) R$601-R$800 ( ) R$801-R$1000

( ) R$1001-R$1200 ( ) R$1201-R$1400

( ) R$1401-R$1600 ( ) R$1601-R$1800

( ) R$1801-R$2000 ( ) R$2001-R$2500

( ) R$2501-R$3000 ( ) R$3001 OU MAIS

**BOLSA FAMÍLIA:**

( ) NÃO SE APLICA ( ) ATÉ R$200

( ) R$201-R$400 ( ) R$401-R$600

( ) R$601-R$800 ( ) R$801-R$1000

( ) R$1001-R$1200 ( ) R$1201-R$1400

( ) R$1401-R$1600 ( ) R$1601-R$1800

( ) R$1801-R$2000 ( ) R$2001-R$2500

( ) R$2501-R$3000 ( ) R$3001 OU MAIS

**VENDA DE ARTESANATO:**

( ) NÃO SE APLICA ( ) ATÉ R$200

( ) R$201-R$400 ( ) R$401-R$600

( ) R$601-R$800 ( ) R$801-R$1000

( ) R$1001-R$1200 ( ) R$1201-R$1400

( ) R$1401-R$1600 ( ) R$1601-R$1800

( ) R$1801-R$2000 ( ) R$2001-R$2500

( ) R$2501-R$3000 ( ) R$3001 OU MAIS

**OUTRAS RENDAS (SEGURO DESEMPREGO, AUXÍLIO MATERNIDADE, PENSÃO ALIMENTÍCIA, VENDA DE PRODUTOS DE AGRICULTURA, CAÇA, COLETA E PESCA, PRODUÇÃO CULTURAL, OUTROS):**

( ) NÃO SE APLICA ( ) ATÉ R$200

( ) R$201-R$400 ( ) R$401-R$600

( ) R$601-R$800 ( ) R$801-R$1000

( ) R$1001-R$1200 ( ) R$1201-R$1400

( ) R$1401-R$1600 ( ) R$1601-R$1800

( ) R$1801-R$2000 ( ) R$2001-R$2500

( ) R$2501-R$3000 ( ) R$3001 OU MAIS

**REPETIR AS MESMAS PERGUNTAS DA PÁGINA 62 PARA CADA MORADOR COM MAIS DE 10 ANOS DE IDADE.**
