# Supplementary material for: Baixo peso ao nascer, prematuridade e restrição de crescimento intra-uterino: resultados dos dados de base da primeira coorte de nascimentos indígenas no Brasil (coorte de nascimentos Guarani)
Source: BMC Pregnancy Childbirth. 2020 Dec 2;20:748. doi: 10.1186/s12884-020-03396-8 (PMC7709282; doi:10.1186/s12884-020-03396-8)
Supplement: Supplementary file 2 — Additional file 2 Supplementary file 2. The Guarani Birth Cohort - Perinatal Questionnaire English version (translated). The Perinatal questionnaire comprised 102 questions and some dependent subquestions, that were organized in blocks to be completed during the interview with the mother (33 items). Some information was extracted from secondary registries (69 items), to avoid unnecessarily long interviews for the participant. The total time for completing the questionnaire was about an hour, including a 30-min interview. The questionnaire was designed in digital format, operated on a handheld personal digital assistant (PDA), with subsequent online data transmission to the study coordinators. The perinatal questionnaire was designed by the research team and was based on questionnaires from the National Census, the First National Survey of Indigenous People’s Health and Nutrition in Brazil, a population-based case-control study in the Guarani population, and other Brazilian reference studies on infant-maternal health, such as the four Pelotas Birth Cohorts (1982, 1993, 2004 and 2015) and the Birth in Brazil Study. We also added questions based on the forms routinely used in health services for prenatal care, the hospital discharge summary after birth, the health card of the child, and the birth certificate. [file 12884_2020_3396_MOESM2_ESM.pdf]

GUARANI BIRTH COHORT

Standardized perinatal data collection instrument

(The perinatal instrument was designed in digital format, and operated on a handheld personal digital assistant (PDA), with subsequent online data transmission to the study coordinators. This Supplementary file was provided in order to inform which data was collected)

THE STANDARDIZED PERINATAL INSTRUMENT SHOULD BE APPLIED TO ALL VILLAGE RESIDENT WOMEN ELEGIBLE FOR THE STUDY, IN THE POSTPARTUM OR POST-ABORTION PERIOD. THE INSTRUMENT SHOULD BE APPLIED AS SOON AS POSSIBLE, PREFERABLY IN THE FIRST FIFTEEN DAYS (TWO WEEKS) AFTER GIVING BIRTH OR ABORTION. IN CASE OF TWIN PREGNANCY, PLEASE, APPLY AN INSTRUMENT FOR ONE OF EACH CHILD AND INDICATE NEWBORN 1 AND NEWBORN 2 OF THE SAME MOTHER. IF YOU HAVE ANY QUESTION, PLEASE, CONTACT THE RESEARCH TEAM COORDINATION IMMEDIATLY.

SECTION A - CHILD IDENTIFICATION VARIABLES

P1 – INTERVIEW DATE: ______/________/________ (DD/MM/YYYY)

INTERVIEWER'S NAME: ____

FULL MOTHER'S NAME: ____

MOTHER'S MARITAL STATUS:
( ) WITH HUSBAND/PARTNER ( ) WITHOUT HUSBAND/PARTNER

P2 – MOTHER'S BIRTH DATE: ___/____/_______ (DD/MM/YYYY) (If there is this data, jump to Page 4 (P4). If not, go to P3 – instruction for the programmer).

P3 – MOTHER´S AGE (only if there is no mother's birth date): ______ YEARS.

P4 – "POLO BASE" OF RESIDENCE: ____

VILLAGE: ______________________________________

P5 - THE MOTHER CAN BE INTERVIEWED? ( ) YES ( ) NO.

IF YES → JUMP TO **P9 (**instruction for the programmer)
IF NO →JUMP TO **P6 (**instruction for the programmer)

P6 – WHY SHE CANNOT BE INTERVIEWED?
( ) REFUSAL ( ) DEATH ( ) HOSPITALIZED WITH RESTRICTION TO INTERVIEW
( ) OTHERS

IF REFUSAL → END OF INTERVIEW. **(**instruction for the programmer)
IF ALL OTHER OPTIONS, → GO TO P7. **(**instruction for the programmer)

P7 – THE INTERVIEW CAN BE DONE WITH ANOTHER RELATIVE OR INFORMANT?
( ) YES ( ) NO, REFUSAL ( ) NO, NOBODY WAS AVAILABLE ( ) NO, OTHERS

IF NO (REFUSAL OR NO AVAILABILITY) → END OF INTERVIEW. **(**instruction for the programmer)

P8 – DEGREE OF RELATIONSHIP BETWEEN INFORMANT AND CHILD'S MOTHER?
( ) PARENTS ( ) PARTNER OR HUSBAND ( ) SON OR DAUGHTER ( ) OTHER

SECTION B – DEMOGRAPHIC AND E SOCIOECONOMIC HOUSEHOLD CHACARCTERISTICS

P9 OPEN FIRE USE? ( ) YES ( ) NO. IF NO → P10 **(**instruction for the programmer)

IF YES, WHAT IS **THE LOCATION OF THIS FIRE?** ( ) INDOOR ( ) OUTSIDE HOUSE

IF INDOOR**, WHERE IS ITS LOCATION?**
( ) SINGLE ROOM ( ) KITCHEN ( ) BEDROOM ( ) LIVING ROOM ( ) OTHER

IF OUTSIDE HOUSE**, WHERE IS ITS LOCATION?**
( ) ON THE GROUND (BONEFIRE) ( ) OUTDOOR KITCHEN, CLOSED
( ) OUTDOOR KITCHEN, WITHOUT WALLS ( ) BALCONY, WITHOUT WALLS ( ) OTHER

P10 WOOD BURNING STOVE USE? ( ) YES ( ) NO.

IF NO → **P11 (**instruction for the programmer)

IF YES, WHAT IS **THE LOCATION OF THIS FIRE?** ( ) INDOOR ( ) OUTSIDE HOUSE

IF INDOOR**, WHERE IS ITS LOCATION?**
( ) SINGLE ROOM ( ) KITCHEN ( ) BEDROOM ( ) LIVING ROOM ( ) OTHER

IF OUTSIDE HOUSE**, WHERE IS ITS LOCATION?**
( ) ON THE GROUND (BONEFIRE) ( ) OUTDOOR KITCHEN, CLOSED
( ) OUTDOOR KITCHEN, WITHOUT WALLS ( ) BALCONY, WITHOUT WALLS ( ) OTHER

P11 GAS STOVE USE? ( ) YES ( ) NO.

IF NO → P12 **(**instruction for the programmer)
IF YES, WHAT IS **THE LOCATION OF THIS FIRE?** ( ) INDOOR ( ) OUTSIDE HOUSE

IF INDOOR**, WHERE IS ITS LOCATION?**
( ) SINGLE ROOM ( ) KITCHEN ( ) BEDROOM ( ) LIVING ROOM ( ) OTHER

IF OUTSIDE HOUSE**, WHERE IS ITS LOCATION?**
( ) ON THE GROUND (BONEFIRE) ( ) OUTDOOR KITCHEN, CLOSED
( ) OUTDOOR KITCHEN, WITHOUT WALLS ( ) BALCONY, WITHOUT WALLS ( ) OTHER

P12 – WHICH FIRE IS MOST USED FOR COOKING?
( ) OPEN FIRE ( ) WOOD BURNING STOVE ( ) GAS STOVE ( ) OTHER

P13 - WHERE THE RESIDENTS OF THE HOUSE USED TO EVACUATE OR DEFECATE?
( ) INDOOR HOUSEHOLD FACILITY
( ) OUTDOOR HOUSEHOLD FACILITY, USED ONLY BY HOUSEHOLD RESIDENTS
( ) OUTDOOR HOUSEHOLD FACILITY, COLLECTIVE (NOT EXCLUSIVE OF THE HOUSEHOLD RESIDENTES)
( ) RUDIMENTARY PIT LATRINE ( ) OUTDOORS IN THE OPEN ( ) OTHER
( ) IGNORED

IF OUTDOORS IN THE OPEN, OTHER OR IGNORED → P14 (instruction for the programmer)

IF ONE OF ANY FACILITY OPTIONS OR RUDIMENTARY PIT LATRINE: WHAT IS THE DESTINATION OF HUMAN WASTE?
( ) PUBLIC SEWAGE DISPOSAL SYSTEM ( ) LOCAL SEPTIC SYSTEM (FUNASA, SESAI, OR OTHER) ( ) RUDIMENTARY PIT ( ) IN THE OPEN
( ) RIVER, LAKE, OR OCEAN ( ) OTHER ( ) IGNORED

P14 WHAT IS THE PREDOMINANT POINT OF COLLECTING WATER FOR GENERAL USE IN THE HOUSEHOLD?
( ) TAP OR SPOUT INSIDE HOME
( ) TAP OR SPOUT OUTSIDE HOME, USED ONLY BY HOUSEHOLD RESIDENTS
( ) TAP OR SPOUTOUTSIDE HOME, COLLECTIVE (NOT EXCLUSIVE OF THE HOUSEHOLD RESIDENTES)
( ) SPRING OR WELL IN THE VILLAGE
( ) WATERFALL, RIVER, OR LAKE
( ) OTHER ( ) IGNORED

IF DIRECT WATERFALL, RIVER, OR LAKE, OR OTHER → **P15** (instruction for the programmer)

IF ONE OF THE TAP OR SPOUT OPTIONS: WHAT IS THE SOURCE OF WATER FOR THE TAP OR SPOUT?
( ) MUNICIPAL SYSTEM
( ) LOCAL SYSTEM (SPRING OR ARTESIAN WELL) (FUNASA, SESAI, OR OTHER)
( ) SPRING OR SHALLOW WELL IN THE VILLAGE
( ) WATERFALL, RIVER, OR LAKE
( ) OTHER
( ) IGNORED

P15 – SOURCE OF DRINKING WATER?

( ) TAP OR SPOUT INSIDE HOME
( ) TAP OR SPOUT OUTSIDE HOME, USED ONLY BY HOUSEHOLD RESIDENTS
( ) TAP OR SPOUTOUTSIDE HOME, COLLECTIVE (NOT EXCLUSIVE OF THE HOUSEHOLD RESIDENTES)
( ) SPRING OR WELL IN THE VILLAGE
( ) WATERFALL, RIVER, OR LAKE
( ) OTHER ( ) IGNORED

DO YOU USUALLY STORE WATER TO DRINK AT HOME? ( ) YES ( ) NO

DO YOU USUALLY TREAT STORED WATER BEFORE DRINKING? (CHLORINE, SODIUM HYPOCLORITE, FILTERED, BOILED, OR LEACHED)?
( ) YES ( ) NO ( ) IGNORED

P16 – WHAT IS THE DESTINATION OF THE HOUSEHOLD TRASH?
( ) COLLECTED BY PUBLIC REMOVAL SERVICE IN THE HOUSEHOLD
( ) STORED IN COLLECTIVE GARBAGE, PERIODICALLY EMPTIED BY PUBLIC REMOVAL SERVICE
( ) BURNED, BURIED, OR DISCARDED IN THE VILLAGE
( ) OTHER ( ) IGNORED

P17 - IS THERE ELECTRICITY IN THE HOME?
( ) YES ( ) NO

IF YES, WHAT IS THE SOURCE OF ELECTRICITY**?**
( ) ELECTRICAL NETWORK OF DISTRIBUTING COMPANY ( ) POWER GENERATOR
( ) SOLAR POWER ( ) OTHER ( ) IGNORED

P18 – DO YOU HAVE:

RADIO? ( ) YES ( ) NO
IF YES, HOW MANY? _____

REFRIGERATOR? ( ) YES ( ) NO
IF YES, HOW MANY? _____

FREEZER? ( ) YES ( ) NO
IF YES, HOW MANY? _____

DVD/BLUE RAY? ( ) YES ( ) NO
IF YES, HOW MANY? _____

GAS STOVE? ( )YES ( ) NO
IF YES, HOW MANY? _____

P19 - DO YOU HAVE:

WATER FILTER / PURIFIER? ( ) YES ( ) NO
IF YES, HOW MANY? _____

WASHING MACHINE? ( ) YES ( ) NO
IF YES, HOW MANY? _____

MICROWAVE / ELECTRIC OVEN? ( ) YES ( ) NO
IF YES, HOW MANY? _____

LAND PHONE? ( ) YES ( ) NO
IF YES, HOW MANY? _____

P20 -DO YOU HAVE:

MOBILE PHONE ( ) YES ( ) NO
IF YES, HOW MANY? _____

COMPUTER/TABLET? ( ) YES ( ) NO
IF YES, HOW MANY? _____

MOTORCYCLE? ( ) YES ( ) NO
IF YES, HOW MANY? _____

TRACTION ANIMAL? ( ) YES ( ) NO
IF YES, HOW MANY? _____

P21 -DO YOU HAVE:

TELEVISION? ( ) YES ( ) NO
IF YES, HOW MANY? _____

CAR? ( ) YES ( ) NO
IF YES, HOW MANY? _____

SATELLITE DISH? ( ) YES ( ) NO
IF YES, HOW MANY? _____

BICYCLE? ( ) YES ( ) NO
IF YES, HOW MANY? _____

P22 - INDICATE THE SOURCES OF THE FOOD CONSUMED AT YOUR HOME IN THE LAST MONTH (MULTIPLE ANSWER):
( ) PLANTATION OR BREEDING OF ANIMALS
( ) HUNTING AND FISHING
( ) FOOD COLLECTION
( ) EXCHANGE OR DONATION INSIDE THE VILLAGE
( ) DONATIONS FROM OUTSIDE THE VILLAGE
( ) PURCHASE

P23 – WHAT IS THE MAIN FOOD SOURCES IN THE HOUSEHOLD?
( ) PLANTATION OR BREEDING OF ANIMALS
( ) HUNTING AND FISHING
( ) FOOD COLLECTION
( ) EXCHANGE OR DONATION INSIDE THE VILLAGE
( ) DONATIONS FROM OUTSIDE THE VILLAGE
( ) PURCHASE

DID THE FAMILY RECEIVE BASIC BASKETS LAST MONTH?
( ) YES ( ) NO ( ) IGNORED

IF NOT OR IGNORED ⟶ **P24 (**instruction for the programmer)

IF YES, **HOW MANY? ____**

P24 WHAT WAS THE HIGHEST LEVEL OF MOTHER'S FORMAL EDUCATION? (YEARS OF STUDY)
( ) Never studied
( ) Studied, but does not know how to inform the level of education
( ) Studied, but no year has been completed
( ) 1st year – elementary school
( ) 2nd year – elementary school
( ) 3rd year – elementary school
( ) 4th year – elementary school
( ) 5th year – elementary school
( ) 6th year – elementary school
( ) 7th year – elementary school
( ) 8th year – elementary school
( ) 9th year – elementary school
( ) 1st year – high school
( ) 2nd year – high school
( ) 3rd year – high school
( ) University Graduate
( ) Postgraduate Studies

SECTION C: ABOUT GESTATION, DELIVERY, POSTPARTUM AND NEWBORN (INTERVIEW)

P25 – CHILDBIRTH LOCATION?
( ) VILLAGE ( ) HOSPITAL ( ) SUPPORT HOUSE FOR INDIGENOUS PATIENTS OUTSIDE THE VILLAGE (CASAI) ( ) OTHER ( ) IGNORED

WHO CARRIED OUT THE DELIVERY?
(**)** DOCTOR ( ) NURSE ( ) MIDWIFE ( ) INDIGENOUS HEALTH AGENT
( ) DELIVERY WITHOUT AID (ALONE) ( ) OTHER ( ) IGNORED

P26 THE PREGNANCY WAS FOLLOWED BY AN INIDIGENOUS MIDWIFE??
( ) YES ( ) NO ( )IGNORED

P27 – DID THE MOTHER SMOKE INDUSTRIALIZED CIGARETTE DURING PREGNANCY?
( ) YES ( ) NO ( ) IGNORED

IF NOT OR IGNORED → **P29 (**instruction for the programmer)

IF YES, **DID THE MOTHER SMOKE DURING ALL PREGNANCY?**
( ) YES ( ) NO ( )IGNORED

IF YES → **P28 (**instruction for the programmer)

IF NO, OR IGNORED, **DID THE MOTHER SMOKE DURING THE FIRST TRIMESTER OF PREGNANCY?**
( ) YES ( ) NO ( )IGNORED

IF NO, OR IGNORED, **DID THE MOTHER SMOKE DURING THE SECOND TRIMESTER OF PREGNANCY?**
( ) YES ( ) NO ( )IGNORED

IF NO, OR IGNORED, **DID THE MOTHER SMOKE DURING THE THIRD TRIMESTER OF PREGNANCY?**
( ) YES ( ) NÃO ( ) IGNORED

P28 - IN THE SMOKING PERIOD DURING PREGNANCY, THE MOTHER SMOKED:

( ) DAILY ( ) WEEKLY, BUT NOT EVERY DAY ( ) MONTHLY, BUT NOT EVERY WEEK ( ) EVENTUALLY ( ) IGNORED

HOW MANY CIGARETTES DOES THE MOTHER SMOKED A DAY DURING PREGNANCY? (ONE PACK = 20 CIGARETTES): _____

P29 – DID THE MOTHER DRINK ANY ALCOHOLIC BEVERAGE DURING PREGNANCY?
( )YES ( ) NO ( )IGNORARED

IF NO, OR IGNORED →**P32 (**instruction for the programmer)

IF YES, **DID THE MOTHER DRINK DURING ALL PREGNANCY?**
( ) YES ( ) NO ( ) IGNORED

IF YES → **P31 (**instruction for the programmer)

IF NO, OR IGNORED → **P30 (**instruction for the programmer)

P30 – DID THE MOTHER DRINK ALCOHOLIC BEVERAGE DURING THE FIRST TRIMESTER OF PREGNANCY?
( ) YES ( ) NO ( )IGNORED

DID THE MOTHER DRINK ALCOHOLIC BEVERAGE DURING THE SECOND TRIMESTER OF PREGNANCY?
( ) YES ( ) NO ( )IGNORED

DID THE MOTHER DRINK ALCOHOLIC BEVERAGE DURING THE THIRD TRIMESTER OF PREGNANCY?
( ) YES ( ) NO ( )IGNORED

P31- WHAT WAS THE MAIN TYPE OF ALCOHOLIC BEVERAGE CONSUMED DURING PREGNANCY?
( ) WINE ( ) BEER ( ) DISTILLED BEVERAGE (SUGARCANE LIQUOR, VODKA, WHISKY, RUM)

IN THE DRINKING PERIOD DURING PREGNANCY, THE MOTHER DRUNK:
( ) ONCE A MONTH OR LESS ( ) 2 TO 4 TIMES A MONTH ( ) 2 OR 3 TIMES A WEEK
( ) 4 TIMES OR MORE A WEEK ( ) IGNORED

ABOUT THE NEWBORN

P32
AT WHICH AGE WAS THE CHILD BREASTFED BY THE MOTHER FOR THE FIRST TIME?
( ) IN THE 1ST HOUR OF LIFE ( ) AFTER THE 1ST HOUR OF LIFE UNTIL 24 HOURS
( ) 2ND DAY ( ) 3RD DAY ( ) 4TH DAY ( ) 5TH DAY ( ) 6TH DAY ( ) 7TH DAY
( ) 8TH DAY OR MORE ( ) NOT YET BREASTFED

WAS THE CHILD BREASTFED BY ANOTHER WOMAN?
( ) YES ( ) NO ( ) IGNORED

IF YES, AT WHICH AGE WAS THE CHILD BREASTFED BY THE ANOTHER WOMAN FOR THE FIRST TIME?
( ) IN THE 1ST HOUR OF LIFE ( ) AFTER THE 1ST HOUR OF LIFE UNTIL 24 HOURS
( ) 2ND DAY ( ) 3RD DAY ( ) 4TH DAY ( ) 5TH DAY ( ) 6TH DAY ( ) 7TH DAY
( ) 8TH DAY OR MORE ( ) NOT YET BREASTFED

WAS THE CHILD KEPT BREASTFED BY THE MOTHER WHILE WAS BEING BREASTFED BY ANOTHER WOMAN?

( ) YES ( ) NO ( )IGNORED

P33 - HAS THE CHILD ALREADY DRANK WATER, TEA, FRUIT JUICE OR ANY TRADITIONAL DRINK? ( ) YES ( ) NO ( )IGNORED

HAS THE CHILD ALREADY DRUNK MILK POWDER, COW'S MILK, GOAT'S MILK, OR OTHER NON-HUMAN MILK?
( ) YES ( ) NO ( )IGNORED

P34 - HAVE THE CHILD ALREADY USED BOTTLE? ( ) YES ( ) NO ( )IGNORED

HAVE THE CHILD ALREADY USED PACIFIER? ( ) YES ( ) NO ( )IGNORED

DOES THE CHILD'S FATHER HAVE ASTHMA? ( ) YES ( ) NO ( )IGNORED

DOES THE CHILD'S MOTHER HAVE ASTHMA? ( ) YES ( ) NO ( )IGNORED

P35 – CHARACTERISTICS OF THE HOUSEHOLD

CHECK THE TYPE OF PREDOMINANT FLOOR:
( ) GROUND ( ) WOOD ( ) CEMENT ( ) CERAMIC ( ) OTHER ( ) IGNORED

CHECK IF ANOTHER TYPE OF MATERIAL IS USED TO COVER THE FLOOR:
( ) NONE ( ) GROUND ( ) WOOD ( ) CEMENT ( ) CERAMIC
( ) PLASTIC, CANVAS ( ) OTHER ( ) IGNORED

CHECK THE TYPE OF PREDOMINANT WALL:
( ) PALM THATCH ( ) STEM ( ) WOODEN BOARD ( ) COB WALL ( ) MASONRY
( ) PLASTIC, CANVAS ( ) SHEET, BLANKET, CLOTH ( ) OTHER ( ) IGNORED

CHECK IF ANOTHER TYPE OF MATERIAL IS USED TO COVER THE WALL:
( ) PALM THATCH ( ) STEM ( ) WOODEN BOARD ( ) COB WALL ( ) MASONRY
( ) PLASTIC, CANVAS ( ) SHEET, BLANKET, CLOTH ( ) OTHER ( ) IGNORED

CHECK THE TYPE OF PREDOMINANT ROOFING:
( ) PALM THATCH ( ) STEM ( ) WOOD CHIPS ( ) WOODEN BOARD
( ) CEMENT SLAB ( ) CLAY TILE ( ) ZINC TILE ( ) PLASTIC, CANVAS
( ) OTHER ( ) IGNORED

P36 - CHECK IF ANOTHER TYPE OF MATERIAL IS USED TO COVER THE ROOFING:
( ) PALM THATCH ( ) STEM ( ) WOOD CHIPS ( ) WOODEN BOARD
( ) CEMENT SLAB ( ) CLAY TILE ( ) ZINC TILE ( ) PLASTIC, CANVAS
( ) OTHER ( ) IGNORED

HOW IS THE INTERNAL HOUSEHOLD DIVISION?
( ) SINGLE ROOM WITHOUT BALCONY ( ) SINGLE ROOM WITH BALCONY
(MORE THAN ONE ROOM WITHOUT BALCONY ( ) MORE THAN ONE ROOM WITH BALCONY

IF SINGLE ROOM → **P38 (**instruction for the programmer)
IF MORE THAN SINGLE ROOM → **P37 (**instruction for the programmer)

P37
HOW MANY BEDROOMS? ________

HOW MANY LIVING ROOMS? ________

HOW MANY BATHROOMS? ________

HOW MANY KITCHENS? ________

HOW MANY EXTERNAL DOORS ARE THERE IN THE HOUSE? ________

HOW MANY WINDOWS ARE THERE IN THE HOUSE?

SECONDARY DATA: these secondary data should be extracted from the prenatal registry card, maternal medical chart, death certificate (DO), birth declaration (DNV), child's card, or other available data sources in the health facility.

P38
TYPE OF PREGNANCY: ( ) SINGLE ( ) TWIN

IF TWIN, **HOW MANY TWINS? _______**

PREGNANCY´S OUTCOME: ( ) LIVE BIRTH ( ) STILLBIRTH ( ) ABORTION

P39 - DID THE MOTHER HAVE ANY PRENATAL CONSULTATION IN THIS PREGNANCY?
( ) YES ( ) NO ( ) IGNORED
IF NO, OR IGNORED →P56 **(**instruction for the programmer)

INDICATE THE SOURCES USED TO EXTRACT PRENATAL AND CHILD DATA (ALLOWS MULTIPLE ANSWER):
( ) PRENATAL REGISTRY CARD
( ) MATERNAL AND CHILD MEDICAL CHARTS
( )CHILD´S VACCINATION CARD
( ) CHILD'S HEALTH CARD
( ) MOTHER´S HEALTH CARD
( ) MULTIDISCIPLINARY INDIGENOUS HEALTH TEAM OWN RECORDS
( ) DEATH CERTIFICATE
( ) BIRTH DECALRATION
( ) OTHER SOURCE

WHERE THE MOTHER DID MOST OF THE PRENATAL CONSULTATIONS?
( ) VILLAGE ( ) OUTSIDE THE VILLAGE
( ) BOTH IN THE VILLAGE AND OUTSIDE THE VILLAGE.

IF IN THE VILLAGE → **P40 (**instruction for the programmer)
IF OUTSIDE OF THE VILLAGE**, WHERE? ____**

P40 -DATE OF LAST MENSTRUATION (LMD): ____/_____/_________. (DD/MM/YYYY)

OBSTETRIC ULTRASOUND (USG)
( ) REQUESTED AND DONE ( ) REQUESTED, BUT NOT DONE
( ) REQUESTED, BUT NO INFOMRMATION ABOUT IF IT WAS DONE OR NOT
( ) NOT REQUESTED ( ) IGNORED

DATE OF THE 1ST ULTRASOUND DURING PREGNANCY ____/_____/______.(DD/MM/YYYY)

GESTATIONAL AGE IN WEEKS, IN THE FIRST PRENATAL CONSULTATION (LMD, USG OR UTERINE HEIGHT):_______

HOW MANY PRENATAL CONSULTATIONS DURING PREGNANCY (WITH DOCTOR OR NURSE): _____

OBSTETRIC HISTORY

P41
NUMBER OF PREGNANCIES (INCLUDING THE LAST PREGNANCY): _____

NUMBER OF ABORTIONS (BEFORE THE LAST PREGNANCY):____

NUMBER OF BIRTHS (BEFORE THE LAST PREGNANCY): _____

HOW MANY LIVE BORNES (BEFORE THE LAST PREGNANCY)?_____

HOW MANY STILLBIRTHS (BEFORE THE LAST PREGNANCY)? _____

P42
MOST RECENT MOTHER´S WEIGHT BEFORE PREGNANCY (KG). ____,____

DATE IN WHICH THE MOTHER´S MOST RECENT WEIGHT WAS MEASURED BEFORE PREGNANCY ____/_____/______

LAST MOTHER´S WEIGHT DURING THE PREGNANCY (KG). ____,____

DATE IN WHICH THE MOTHER´S WEIGHT WAS MEASURED FOR THE LAST TIME DURING PREGNANCY ____/_____/______.

P43
MOST RECENT MOTHER´S HEIGHT (CM) _______ ,___ .

DATE IN WHICH THE MOTHER´S MOST RECENT HEIGHT WAS MEASURED ____/_____/_________.

P44
WAS THERE A PRESCRIPTION REGISTRY OF FERROUS SULPHATE (AT LEAST 1 TABLET PER DAY) FROM THE FIRST PRENATAL CONSULTATION UNTIL THE END OF PREGNANCY?
( ) YES ( ) NO ( )IGNORED

MONTH OF FIRST PRESCRIPTION OF FERROUS SULFATE: ____.
NUMBER OF MONTHS OF PREGNANCY COVERED WITH PRESCRIPTION OF FERROUS SULFATE: ____.

P45

PRENATAL REQUESTED EXAMS

BLOOD TYPE (ABO) + Rh:
( ) REQUESTED AND DONE ( ) REQUESTED, BUT NOT DONE
( ) REQUESTED, BUT NO INFOMRMATION ABOUT IF IT WAS DONE OR NOT
( ) NOT REQUESTED ( ) IGNORED

BLOOD COUNT:
( ) REQUESTED AND DONE ( ) REQUESTED, BUT NOT DONE
( ) REQUESTED, BUT NO INFOMRMATION ABOUT IF IT WAS DONE OR NOT
( ) NOT REQUESTED ( ) IGNORED

FASTING BLOOD GLUCOSE:
( ) REQUESTED AND DONE ( ) REQUESTED, BUT NOT DONE
( ) REQUESTED, BUT NO INFOMRMATION ABOUT IF IT WAS DONE OR NOT
( ) NOT REQUESTED ( ) IGNORED

SYPHILIS TESTING (Venereal Disease Research Laboratory - VDRL):
( ) REQUESTED AND DONE ( ) REQUESTED, BUT NOT DONE
( ) REQUESTED, BUT NO INFOMRMATION ABOUT IF IT WAS DONE OR NOT
( ) NOT REQUESTED ( ) IGNORED

URINE 1:
( ) REQUESTED AND DONE ( ) REQUESTED, BUT NOT DONE
( ) REQUESTED, BUT NO INFOMRMATION ABOUT IF IT WAS DONE OR NOT
( ) NOT REQUESTED ( ) IGNORED

URINOCULTURE:
( ) REQUESTED AND DONE ( ) REQUESTED, BUT NOT DONE
( ) REQUESTED, BUT NO INFOMRMATION ABOUT IF IT WAS DONE OR NOT
( ) NOT REQUESTED ( ) IGNORED

RAPID TEST OR ELISA TEST FOR HIV:
( ) REQUESTED AND DONE ( ) REQUESTED, BUT NOT DONE
( ) REQUESTED, BUT NO INFOMRMATION ABOUT IF IT WAS DONE OR NOT
( ) NOT REQUESTED ( ) IGNORED

P46
HBsAg (HEPATITIS B ANTIGEN S):
( ) REQUESTED AND DONE ( ) REQUESTED, BUT NOT DONE
( ) REQUESTED, BUT NO INFOMRMATION ABOUT IF IT WAS DONE OR NOT
( ) NOT REQUESTED ( ) IGNORED

TOXOPLASMOSIS SOROLOGY:
( ) REQUESTED AND DONE ( ) REQUESTED, BUT NOT DONE
( ) REQUESTED, BUT NO INFOMRMATION ABOUT IF IT WAS DONE OR NOT
( ) NOT REQUESTED ( ) IGNORED

FECAL (STOOL) EXAM/OVA AND PARASITE TEST :
( ) REQUESTED AND DONE ( ) REQUESTED, BUT NOT DONE
( ) REQUESTED, BUT NO INFOMRMATION ABOUT IF IT WAS DONE OR NOT
( ) NOT REQUESTED ( ) IGNORED

P47
HAS THE MOTHER RECEIVED AT LEAST 3 DOSES OF TETANUS VACCINE BEFORE THE LAST DELIVERY?
( ) YES ( ) NO ( )IGNORED

HAS THE MOTHER RECEIVED AT LEAST A DOSE OF DIPHTHERIA AND TETANUS VACCINE (dT) IN THE LAST FIVE YEARS?
( ) YES ( ) NO ( )IGNORED

HAS THE MOTHER RECEIVED AT LEAST 3 DOSES OF HEPATITIS B VACCINE BEFORE THE LAST DELIVERY?
( ) YES ( ) NO ( )IGNORED

HAS THE MOTHER UNDERGONE ODONTOLOGIC EXAM DURING LAST PREGNANCY?
( ) YES ( ) NO ( )IGNORED

P48
DURING PREGNANCY, THERE WAS REGISTRATION OF:
BLOOD PRESSURE? **( )** YES ( ) NO.
IF YES, **HOW MANY REGISTRIES? ______**

BODY HEIGHT? **( )** YES ( ) NO.
IF YES, **HOW MANY REGISTRIES? ______**

BODY WEIGHT? ( ) YES ( ) NO.
IF YES, **HOW MANY REGISTRIES? ______**

P49
(UTERINE) FUNDAL HEIGHT? ( ) YES ( ) NO.
IF YES, **HOW MANY REGISTRIES? ______**

FETAL HEART RATE? **( )** YES ( ) NO.
IF YES, **HOW MANY REGISTRIES? ______**

BREAST EXAMINATION? **( )** YES ( ) NO.
IF YES, **HOW MANY REGISTRIES? ______**

P50
WAS THE HIV RAPID TEST DONE ON MOTHER DURING LABOR?
( ) YES ( ) NO ( )IGNORED

WAS THE SYPHILIS RAPID TEST DONE ON MOTHER DURING LABOR?
( ) YES ( ) NO ( )IGNORED

TYPE OF DELIVERY ( ) VAGINAL ( ) CESAREAN
If vaginal → **P52 (**instruction for the programmer)

P51

WHAT WAS THE CLINICAL INDICATION FOR CESAREAN SECTION?

( ) FETAL DISTRESS
( ) CEPHALOPELVIC DISPROPORTION
( ) PRESENTATION DISORDERS
( ) MATERNAL BLEEDING
( ) FAILURE OF PROGRESSION
( ) ECLAMPSIA
( ) PRE-ECLAMPSIA
( ) POST-MATURITY
( ) FETAL DEATH
( ) GESTATIONAL DIABETES
( ) PREVIOUS C-SECTION
( ) FAMALE STERILIZATION
( ) OTHER

P52

INDICATE IF OCCURRED DURING LABOR: (MULTIPLE CHOICE)

( ) INDUCTION (OXYTOCIN)
( ) ANALGESIA
( ) EPISIOTOMY
( ) FÓRCEPS
( ) NONE OF THE EVENTS ABOVE

P53

INDICATE WHICH OF THESE EVENTS TAKE PART OF THE PREVIOUS PATHOLOGICAL HISTORY AND INTERCORRENCES DURING PREGNANCY AND DELIVERY (ALLOWS MULTIPLE CHOICE).

( ) PREGESTATIONAL HYPERTENSION
( ) GESTATIONAL HYPERTENSION
( ) PRE-ECLAMPSIA
( ) ECLAMPSIA
( ) CARDIOPATIHY
( ) PREGESTATIONAL DIABETES
( ) GESTATIONAL DIABETES
( ) NONE OF THE EVENTS ABOVE

P54

( ) URINARY INFECTION IN PREGNANCY
( ) RISK OF PRETERM LABOR
( ) CEPHALOPELVIC DISPROPORTION
( ) BLEEDING IN THE FIRST TRIMESTER
( ) BLEEDING IN THE SECOND TRIMESTER
( ) BLEEDING IN THE THIRD TRIMESTER
( ) NONE OF THE EVENTS ABOVE

P55

( ) CHRONIC ANEMIA
( ) PREMATURE RUPTURE OF THE MEMBRANE
( ) NUCHALCORD
( ) NEONATAL ASPHYXIA
( ) MECONIUM ASPIRATION
( ) NONE OF THE EVENTS ABOVE

P56
CHILD'S NAME ____

CHILD´S DATE OF BIRTH: ___/_____/_________. (DD/MM/YYYY)

CHILD'S SEX: ( ) MALE ( ) FEMALE

CHILD´S WEIGHT AT BIRTH (GRAMAS): _________g

CHILD´S LENGTH (CM): _____,__CM

CHILD´S HEAD CIRCUMFERENCE (CM): ______, ___ cm

P57
IS THERE REGISTRY OF GESTATIONAL AGE (CAPURRO)? ( ) YES ( ) NO ( ) IGNORED

INDICATE GESTATIONAL AGE (GA) AT BIRTH: _______WEEKS

WEIGHT / GESTATIONAL AGE RATIO
( ) APPROPRIATE FOR GA ( ) SMALL FOR GA ( ) LARGE FOR GA ( ) IGNORED

APGAR 1': ______. ( ) IGNORED

APGAR 5': _______. ( ) IGNORED

REANIMATION? ( ) YES ( ) NO ( ) IGNORED

P58
OBSERVED PATHOLOGIES (MULTIPLE CHOICE):
( ) HYALINE MEMBRANE
( ) BRONCHOPULMONARY DYSPLASIA
( ) NEONATAL ASPHYXIA
( ) NEUROLOGICAL DISORDERS
( ) BLEEDING
( ) NONE OF THE PATHOLOGIES ABOVE

P59 (MULTIPLE CHOICE)
( ) HYPERBILIRUBINEMIA
( ) CONGENITAL INFECTION
( ) NEONATAL SEPSIS
( ) CONGENITAL ANOMALY
( ) NONE OF THE PATHOLOGIES ABOVE

DID THE NEWBORN NEED NEONATAL INTENSIVE CARE (ICU)?
( ) YES ( ) NO ( ) IGNORED

P60
WAS NEONATAL SCREENING TEST PERFORMED IN NEWBORN?
( ) YES ( ) NO ( ) IGNORED

WAS HEARING SCREENING TEST PERFORMED IN NEWBORN?
( ) YES ( ) NO ( ) IGNORED

WAS EYE SCREENING TEST PERFORMED IN NEWBORN?
( ) YES ( ) NO ( ) IGNORED

THE NEWBORN DIED BEFORE THIS INTERVIEW? ( ) YES ( ) NO
IF YES, the interview is ended and should generate an alert to complete the FINAL questionnaire, concluding the child's participation in the cohort study. **(**instruction for the programmer)

P61
HOW MANY PEOPLE LIVE IN YOUR HOME (INCLUDING THE NEWBORN)? _______.

HOW MANY UNDER FIVE CHILDREN LIVE IN YOUR HOME (INCLUDING THE PARTICIPATING NEWBORN)? _______.

HOW MANY PEOPLE 10 YEARS OLD OR MORE LIVE IN YOUR HOME (SCORE ON SCALE BUTTON)?_____.

THE FOLLOWING QUESTIONS SHOULD BE COMPLETED FOR EACH ONE OF THE RESIDENTS OF THE HOUSE WITH 10 YEARS OLD OR MORE

P62
FIRST NAME: ____

SEX ( ) MALE ( ) FEMALE

AGE: _______ YARS.

DEGREE OF RELATIONSHIP WITH THE NEWBORN:
( ) PARENTS ( ) GRANDPARENTS ( ) BROTHERS ( ) OTHERS

OCCUPATION: ( ) NO OCCUPATION ( ) INDIGENOUS TEACHER
( ) INDIGENOUS HEALTH AGENT (AIS OU AISAN)
( ) SCHOOL COOKER ( ) VIGILANT ( ) BRICKLAYER ( ) NURSE
( ) FARMER
( ) NURSING TECHNICIAN/ENVIRONMENT TECHNICIAN/DENTAL HYGIENE TECHNICIAN
( ) DRIVER ( ) INDIGENOUS FOUNDATION TECHNICIAN ( ) RETIRED ( ) OTHER

LOCAL OF WORK:
( ) VILLAGE ( ) OUTSIDE THE VILLAGE ( ) NOT APPLICABLE

REGULAR INCOME IN THE LAST MONTH (FORMAL EMPLOYMENT WAGE):
( ) Not applicable ( ) UP TO R$200
( ) R$201-R$400 ( ) R$401-R$600
( ) R$601-R$800 ( ) R$801-R$1000
( ) R$1001-R$1200 ( ) R$1201-R$1400
( ) R$1401-R$1600 ( ) R$1601-R$1800
( ) R$1801-R$2000 ( ) R$2001-R$2500
( ) R$2501-R$3000 ( ) R$3001 OR MORE

RETIREMENTS AND PENSIONS (INSS, FUNRURAL, OTHERS):
( ) Not applicable ( ) UP TO R$200
( ) R$201-R$400 ( ) R$401-R$600
( ) R$601-R$800 ( ) R$801-R$1000
( ) R$1001-R$1200 ( ) R$1201-R$1400
( ) R$1401-R$1600 ( ) R$1601-R$1800
( ) R$1801-R$2000 ( ) R$2001-R$2500
( ) R$2501-R$3000 ( ) R$3001 OR MORE

CASH TRANSFER (BOLSA FAMÍLIA):
( ) Not applicable ( ) UP TO R$200
( ) R$201-R$400 ( ) R$401-R$600
( ) R$601-R$800 ( ) R$801-R$1000
( ) R$1001-R$1200 ( ) R$1201-R$1400
( ) R$1401-R$1600 ( ) R$1601-R$1800
( ) R$1801-R$2000 ( ) R$2001-R$2500
( ) R$2501-R$3000 ( ) R$3001 OR MORE

SALE OF HANDICRAFT:

( ) Not applicable	( ) UP TO R$200
( ) R$201-R$400	( ) R$401-R$600
( ) R$601-R$800	( ) R$801-R$1000
( ) R$1001-R$1200	( ) R$1201-R$1400
( ) R$1401-R$1600	( ) R$1601-R$1800
( ) R$1801-R$2000	( ) R$2001-R$2500
( ) R$2501-R$3000	( ) R$3001 OR MORE

OTHER INCOME (UNEMPLOYMENT INSURANCE, MATERNITY ASSISTANCE, FOOD PENSION, SALE OF AGRICULTURE PRODUCTS, HUNTING, COLLECTION AND FISHING, CULTURAL PRODUCTION, OTHERS):

( ) Not applicable	( ) UP TO R$200
( ) R$201-R$400	( ) R$401-R$600
( ) R$601-R$800	( ) R$801-R$1000
( ) R$1001-R$1200	( ) R$1201-R$1400
( ) R$1401-R$1600	( ) R$1601-R$1800
( ) R$1801-R$2000	( ) R$2001-R$2500
( ) R$2501-R$3000	( ) R$3001 OR MORE

REPEAT THE SAME QUESTIONS ON PAGE 62 FOR EVERY RESIDENT OVER 10 YEARS OF AGE.
